# Unveiling impaired vascular function and cellular heterogeneity in diabetic donor-derived vascular organoids

**DOI:** 10.1093/stmcls/sxae043

**Published:** 2024-07-25

**Authors:** Hojjat Naderi-Meshkin, Wiwit A Wahyu Setyaningsih, Andrew Yacoub, Garrett Carney, Victoria A Cornelius, Clare-Ann Nelson, Sophia Kelaini, Clare Donaghy, Philip D Dunne, Raheleh Amirkhah, Anna Zampetaki, Lingfang Zeng, Alan W Stitt, Noemi Lois, David J Grieve, Andriana Margariti

**Affiliations:** The Wellcome-Wolfson Institute for Experimental Medicine, Queen’s University Belfast, Belfast, BT9 7BL, United Kingdom; The Wellcome-Wolfson Institute for Experimental Medicine, Queen’s University Belfast, Belfast, BT9 7BL, United Kingdom; Department of Anatomy, Faculty of Medicine, Public Health, and Nursing, Universitas Gadjah Mada, Sleman, D.I. Yogyakarta, 55281, Indonesia; The Wellcome-Wolfson Institute for Experimental Medicine, Queen’s University Belfast, Belfast, BT9 7BL, United Kingdom; The Wellcome-Wolfson Institute for Experimental Medicine, Queen’s University Belfast, Belfast, BT9 7BL, United Kingdom; The Wellcome-Wolfson Institute for Experimental Medicine, Queen’s University Belfast, Belfast, BT9 7BL, United Kingdom; The Wellcome-Wolfson Institute for Experimental Medicine, Queen’s University Belfast, Belfast, BT9 7BL, United Kingdom; The Wellcome-Wolfson Institute for Experimental Medicine, Queen’s University Belfast, Belfast, BT9 7BL, United Kingdom; The Wellcome-Wolfson Institute for Experimental Medicine, Queen’s University Belfast, Belfast, BT9 7BL, United Kingdom; The Patrick G Johnston Centre for Cancer Research, Queen’s University Belfast, Belfast, BT9 7AE, United Kingdom; The Patrick G Johnston Centre for Cancer Research, Queen’s University Belfast, Belfast, BT9 7AE, United Kingdom; School of Cardiovascular Medicine and Sciences, BHF Centre of Research Excellence, King’s College London, London, SE5 9NU, United Kingdom; School of Cardiovascular Medicine and Sciences, BHF Centre of Research Excellence, King’s College London, London, SE5 9NU, United Kingdom; The Wellcome-Wolfson Institute for Experimental Medicine, Queen’s University Belfast, Belfast, BT9 7BL, United Kingdom; The Wellcome-Wolfson Institute for Experimental Medicine, Queen’s University Belfast, Belfast, BT9 7BL, United Kingdom; The Wellcome-Wolfson Institute for Experimental Medicine, Queen’s University Belfast, Belfast, BT9 7BL, United Kingdom; The Wellcome-Wolfson Institute for Experimental Medicine, Queen’s University Belfast, Belfast, BT9 7BL, United Kingdom

**Keywords:** induced pluripotent stem cells, cardiovascular diseases, diabetic vasculopathy, vascular disease modeling, blood vessel organoids, regenerative medicine

## Abstract

Vascular organoids (VOs), derived from induced pluripotent stem cells (iPSCs), hold promise as in vitro disease models and drug screening platforms. However, their ability to faithfully recapitulate human vascular disease and cellular composition remains unclear. In this study, we demonstrate that VOs derived from iPSCs of donors with diabetes (DB-VOs) exhibit impaired vascular function compared to non-diabetic VOs (ND-VOs). DB-VOs display elevated levels of reactive oxygen species (ROS), heightened mitochondrial content and activity, increased proinflammatory cytokines, and reduced blood perfusion recovery in vivo. Through comprehensive single-cell RNA sequencing, we uncover molecular and functional differences, as well as signaling networks, between vascular cell types and clusters within DB-VOs. Our analysis identifies major vascular cell types (endothelial cells [ECs], pericytes, and vascular smooth muscle cells) within VOs, highlighting the dichotomy between ECs and mural cells. We also demonstrate the potential need for additional inductions using organ-specific differentiation factors to promote organ-specific identity in VOs. Furthermore, we observe basal heterogeneity within VOs and significant differences between DB-VOs and ND-VOs. Notably, we identify a subpopulation of ECs specific to DB-VOs, showing overrepresentation in the ROS pathway and underrepresentation in the angiogenesis hallmark, indicating signs of aberrant angiogenesis in diabetes. Our findings underscore the potential of VOs for modeling diabetic vasculopathy, emphasize the importance of investigating cellular heterogeneity within VOs for disease modeling and drug discovery, and provide evidence of GAP43 (neuromodulin) expression in ECs, particularly in DB-VOs, with implications for vascular development and disease.

## Graphical Abstract

**Graphical Abstract:**
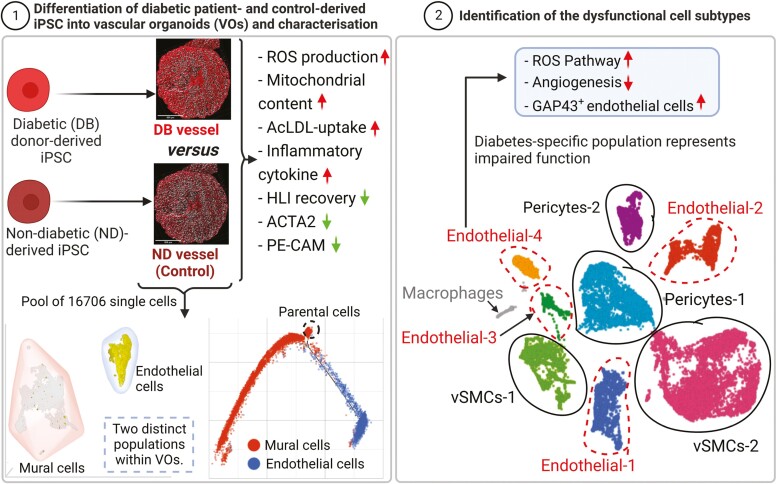

**Significance Statement**

Our study provides a comprehensive single-cell expression atlas of vascular organoids (VOs) and reveals impaired function in diabetic patient-derived organoids arises from a subpopulation of vascular cells, which may represent early signs of aberrant angiogenesis in diabetes. This work highlights the potential of organoids as a platform for disease modeling and drug screening and provides insights for improving the fidelity of VOs as disease models.

## Introduction

Blood vessels play a crucial role in delivering oxygen and nutrients to tissues and organs. Cardiovascular diseases (CVDs) are the leading cause of death globally, with diabetes mellitus (DM) being a significant risk factor. DM is a chronic metabolic disease that affects over 400 million people worldwide and increases the risk of developing CVDs twofold.^1^ Recent studies have provided convincing evidence that DM accelerates endothelial dysfunction, which can become the driving force behind the development and progression of CVDs.^2,3^ Although traditional associations between both diseases have focused on risk factors such as hypertension, obesity, dyslipidemia, and hyperglycemia, current research suggests that alterations in vascular cell composition and function may also contribute to the diseases.

The 2 main cellular components of blood vessels are mural cells (pericytes and vascular smooth muscle cells [VSMCs]) and endothelial cells (ECs), both of which play a crucial role in proper vascular function and are central to pathological dysfunctions.^4,5^ Patient-derived induced pluripotent stem cells (iPSCs)-derived vascular organoids (VOs) are an innovative tool for in vivo and in vitro studies, with the presence of both mural cells and ECs providing new insights into disease-specific phenotypes and biomarkers. Diabetic iPSCs (DB iPSCs) are thought to retain metabolic memory, with VOs derived from these cells being more prone to oxidative stress and dysfunction.^6-9^ However, the cellular composition of iPSCs-derived VOs and how disease-specific phenotypes and biomarkers are displayed in the cell types are not yet fully understood.

This study demonstrates that VOs derived from patients with diabetes can be used to model DB vasculopathy, exhibiting higher levels of proinflammatory cytokines, antiangiogenic proteins, and reactive oxygen species (ROS). Using single-cell RNA sequencing, we identified major vascular cell types and clusters within VOs and uncovered molecular and functional differences as well as signaling networks among these populations. We also discovered that GAP43 is expressed in ECs, particularly in DB-VOs, which could be significant in future studies of vascular development and disease. Our findings emphasize the importance of cellular heterogeneity within VOs for the development of targeted therapies for DM and CVDs.

## Methods

### Experimental design

First, iPSCs were reprogrammed from MNCs of diabetic (DB) and non-diabetic (ND) individuals based on our previously established iPSCs generation and characterization protocol.^10^ The pluripotency capacity of the generated iPSC lines was verified before proceeding to the step of VO generation (Supplementary Figure S1A). Then, iPSCs lines (Table 1) in passage number 12-17 were differentiated into vascular cells in a 3D manner to get diabetic and non-diabetic VOs, called DB-VOs and ND-VOs, respectively. As suggested by previous literature,^9^ to get better disease modeling of DB vasculopathy in vitro, established mature DB-VOs at day 15 from DB iPSCs were started to culture in high glucose concentration (33 mM, adapted from our previous publication^11^), plus 1 ng/mL human TNF [Invitrogen, PHC3011] and 1 ng/mL IL-6 [Invitrogen, PHC3011]). As a control, to maintain the same osmotic pressure in ND-VOs as in DB-VOs, d-mannitol (Sigma-Aldrich) was added to the culture medium of ND-VOs to a final concentration of 33 mM. d-Mannitol is metabolically inert in humans and used as a control of high glucose treatment experiments.^12^ DB-VOs and ND-VOs were then exploited for deeper biological insights interrogated by both wet lab and dry lab analyses. For each analysis, at least 3 biological replicates were used, and experiment-specific details have been mentioned accordingly. Statistical analyses were performed using Prism 9.3.1 (GraphPad) or R 4.1.2. All statistical tests used are described in the figure legends. *P* < .05 or FDR < 0.05 was accepted as statistically significant. The rationale behind selecting, pooling, and using iPSC lines from both DB and ND donors in various experiments is to ensure the robustness and generalizability of our findings. By incorporating multiple iPSC lines from diverse donors representing both DB and ND conditions, we aim to demonstrate that the observed differences are not specific to a particular donor, patient, or a limited number of cell lines. Pooling iPSC lines from various sources helps mitigate potential biases that could arise from individual variations in genetic backgrounds, disease severity, or cellular responses. This approach allows us to capture a broader spectrum of the disease phenotype and its associated variability, thereby strengthening the validity and reliability of our conclusions. Furthermore, by conducting experiments across multiple iPSC lines, we can assess the consistency and reproducibility of our results, enhancing the robustness of our findings. This comprehensive approach enables us to elucidate underlying mechanisms that may be common across DB individuals while also identifying potential inter-individual differences that could inform personalized therapeutic strategies.

### Biological materials and ethical considerations

All methods regarding the human iPSC culture and differentiation had ethical approval from the Office for Research Ethics Committees of Northern Ireland (REC 14/NI/1109) and were performed following the relevant institutional guidelines and regulations including the Declaration of Helsinki and Ethical Guidelines for Medical and Health Research Involving Human Subjects. All experimental procedures of our animal study were performed in accordance with the guidance on the Operation of the Animals (Scientific Procedures) Act, 1986 (UK) and Directive 2010/63/EU of the European Parliament on the protection of animals used for scientific purposes, and were approved by the Queen’s University Belfast Animal Welfare and Ethical Review Body. Work was performed under the project license number PPL2922.

### VO generation

Blood vessel organoids were generated as previously described by Wimmer et al (2019), with some critical modifications that dramatically decrease both labor time and risk of contamination of VOs. Briefly, iPSCs were seeded on a Cell-Matrix (ATCC)-coated 100-mm cell culture dish and expanded for 4 days in StemMACS iPS Brew XF. When iPSCs reached 80%-90% confluence, they were gently detached into cell clusters by 5 minutes incubation in StemMACS Passaging Solution XF. The cell cluster pellet was collected by centrifuge at 600 × *g*, for 5 minutes. The pellet of the iPSC was distributed in small clamps (3-10 cells) into one full ultra-low attachment 6-well plate. The cell aggregates were generated within KO-DMEM plus KO-Serum supplemented with ROCK inhibitor Y-27632 and incubated for only 1 night. It is always critical to evenly distribute the cells/aggregates/spheroids inside Ultra-low attachment 6-well plates just before putting them in 37 °C and 5% CO_2_ incubator.

After reaching a stable aggregate size (>50-200 μm in diameter), the aggregates were precipitated by gravitation for 20 minutes, and mesodermal induction was initiated in N2B27 neurobasal media containing CHIR99021 (12 µM) and BMP-4 (30 ng/mL) for 3 days. For another 2 days, spheroids were transferred into N2B27 neurobasal media containing Forskolin (2 µM) and hVEGF-A (100 ng/mL) to induce the budding of endothelial tubes. We then proceeded to collect the floating spheroids by gravitation and embedded them in a 3D collagen I (PureCol)–Matrigel (Corning) matrix in 12-well plates (6500 µL per well) which allowed the sprouting of vascular networks. 3D collagen I-Matrigel matrix containing homogeneously distributed spheroids was left for 2 hours in a 37 °C and 5% CO_2_ incubator to let it polymerize, and then 1.5 mL of prewarmed StemPro34 (supplemented with VEGF-A [100 ng/mL] and hFGF2 [100 ng/mL] and 15% fetal bovine serum [FBS]) was gently poured onto each matrix from the 12-well wall and incubated for 3 days. From then on, the same media were exchanged every other day. After 5 days in the matrix, the sprouted vascular networks became visible enough to cut every 3-5 of them using a fine-tip, curved tweezer (Supplementary Video S1). The segmented vascular networks were transferred back to the ultra-low attachment 6-well plate gently by a 10-mL serological pipette, to let them self-assemble into mature organoids during the following 5 days. At this stage, they were ready to assess the presence of ECs and pericytes, or for any other experiment.

### Generation of pure human ECs from iPSCs

Spheroids at day 5 of the VO generation protocol were used to derive a pure population of ECs. The spheroids were meticulously sectioned into pieces measuring 100-200 μm in diameter through gentle pipetting with a yellow tip. These sections were then plated onto 100-mm dishes coated with 0.2% gelatin (Sigma-Aldrich). The culture medium for this step consisted of StemPro34 complete medium, supplemented with 1% GlutaMAX, 1% penicillin/streptomycin, 15% FBS (ThermoFisher), 100 ng/mL hVEGF-A (NBC1-21277, Bio-Techne), and 100 ng/mL of FGF-2 (NBP2-34921, Bio-techne). The medium was refreshed once on day 8. By day 10, adherent cells outgrown from small spheroid pieces were magnetically separated using CD144 (VE-Cadherin) MicroBeads (Miltenyi Biotec, 130-097-857) following the manufacturer’s instructions. The isolated CD144+ cells were then seeded onto flasks coated with collagen IV (3410-010-02, Bio-techne) at a concentration of 5 μg/mL. The culture medium, EGM2 complete medium (CC-3202/6, Lonza), was supplemented with 5% FBS, 30 ng/mL hVEGF-A, 20 ng/mL FGF-2, and 10 μM Rock inhibitor (Generon). Achieving confluence within 3-4 days required seeding the cells at a density of 20 000 cells/cm². The purity and identity of the resulting ECs from both DB and ND individuals were validated through flow cytometry using CD144 Antibody (ThermoFisher, Cat No 17-1449-42) and immunostaining with CD31 (ab28364) and CD144 (ab33168; Supplementary Figure S1B-S1E).

### Immunocytochemistry of VOs

VOs (5-10) were transferred into 2-mL microtubes by a wide orifice blue tip (Alpha Laboratories), washed once with Phosphate-buffered saline (PBS), and fixed in paraformaldehyde solution (4%) for 2 hours. Then they were incubated for 3 hours in blocking/permeabilization solution (3% FBS, 1% Bovine serum albumin (BSA) [wt/vol], 0.5% Triton X-100, 0.5% Tween 20, and 0.01% [wt/vol] sodium deoxycholate in PBS^12^), 2 mL per microtube while shaking horizontally on a rocking shaker, after which they were incubated overnight at 4 °C with antibodies: ColIV (ab6311) for basement membrane, CD31 (Ab28364) for ECs and PDGFR-ß (AF385, R&D Systems) for pericytes. Samples were then washed 3 times with PBS and blocked in a 5% donkey serum in PBS solution, followed by washes, adding the appropriate secondary antibodies against the respected host of primary antibodies. incubating and counterstaining with DAPI (Life Technologies, D1306). The stained VOs were mounted on the iSpacers (Sunjin Lab) and mounting media was added at the end of the staining protocol. Samples were covered by a coverslip, examined under a Confocal Microscope (SP8, Leica), and their images were analyzed using ImageJ.

For live staining, VOs were incubated with Calcein-AM (Invitrogen, C3100) for 30 minutes, and immediately mounted on the iSpacers to capture images with Nikon 6D Live Cell Imaging Inverted Microscope within 2 hours.

### Human protein array for both VOs and their secreted media

After 21 days of treatment, protein extracts of VOs were collected and analyzed using the human angiogenesis protein array (RayBiotech, AAH-ANG-1000-8). To use conditioned media for protein array investigation, at 42 days after treatment, 1.5 mL of culture media (minus FBS) was added onto each well of 6-well plates containing 10 organoids, and conditioned media were collected after 24 hours. In total, 4.5 mL mixture of 3 DBs versus 4.5 mL of 3 NDs conditioned media was used for protein quantification, after a 2 minutes centrifugation at high speed to exclude any debris.

Quick Start Bradford protein assay was used to quantify and normalize protein concentration among samples. Additionally, internal positive and negative controls of each array membrane were used to normalize arrays together and subtract signal background, respectively, based on the RayBiotech instruction. Protein array membranes were exposed on chemiluminescence detection, and images were captured by G:BOX (Syngene). Protein Array plugin (Biii) in the ImageJ software was used to normalize and quantify protein levels with pixel intensity on the human protein array membranes containing 43 antibodies.

### ROS production assay

Total ROS Assay Kit 520 nm (Invitrogen, Cat: 88-5930) was used to identify ROS in the cells of VOs by Flow cytometry in the FITC channel as well as confocal imaging. Briefly, VOs were first broken into small pieces with 21-25G needle and then further digested into single cells in DLD for 20 minutes, cultured on 6-well plates for 3-4 days in the same media used for maintaining VOs in culture (StemPro34 medium with its supplements). After removing debris by washing with PBS, the single cells were incubated with the kit for 1 hour as per the manufacturer’s instructions. Immediately, the cells were dissociated using TrypLE Express Enzyme (1X; Thermo Fisher), filtered through pluriStrainer 40 µm MeSH, and subjected to FACS florescent reading in PBS. Some small pieces were kept to image ROS fluorescent immediately by Nikon 6D Live imaging.

### Uptake of acetylated-LDL

The capacity of lipid uptake was monitored using Alexa Fluor 594 Ac-LDL (Invitrogen, L35353). Similar to the ROS assay, dissociated VOs were cultured in a 6-well plate for 3-4 days, and then incubated with 2.5 μg/mL Ac-LDL—Alexa Fluor 594 in StemPro34 medium for 4 hours at 37 °C immediately before confocal or flow cytometry analysis. Thereafter, the cells were dissociated using TrypLE Express Enzyme (1X; Thermo Fisher), filtered through pluriStrainer 40 µm MeSH, and subjected to Flow Cytometry fluorescent reading in PBS. Small pieces of broken organoids were also used for confocal imaging (SP8, Leica) of cellular uptake of Ac-LDL in parallel. Therefore, these VO pieces were further subjected to fixation with 4% PFA for 10 minutes and counterstained with DAPI.

### Measuring mitochondrial content, number, and size

MitoTracker Red is a fluorescent dye taken up by active mitochondria based on their potential. This was used to quantify mitochondrial activity within VOs. VOs were placed in a low-attachment 96-well plate. MitoTracker Red CMXROS (Invitrogen, M7512) was made up to 500 nM in organoid culture media. Organoids were incubated for 1 hour and 30 minutes at 37 °C and 5% CO_2_. The fluorescence was measured using the OMEGA plate reader (average of 20 reads per well). Additionally, the organoids were fixed to image the fluorescence signal as index of mitochondrial activity by confocal imaging.

To investigate mitochondrial number, morphology, and size, VO samples were processed for transmission electron microscopy (TEM). VOs were fixed for 1 hour in 4% paraformaldehyde solution (Thermo Fisher Scientific, 7732-18-5) in PBS and for 2 hours in 2.5% glutaraldehyde in PHEM buffer (TAAB, G016). The PHEM buffer was composed of 5 mM HEPES sodium salt (Sigma-Aldrich, H3784), 60 mM PIPES sodium salt (Calbiochem, 6910), 10 mM EGTA (EDM Millipore, 324626), 2 mM magnesium chloride (Sigma-Aldrich, M4880). The Leica EMTP automatic tissue processor was used to process the VOs. The samples were washed in PHEM buffer and fixed in 1% osmium tetroxide (TAAB, O016). Serial dilutions of ethanol (Sigma-Aldrich, 51976) and Spurr low viscosity embedding kit (Electron Microscopy Sciences, 22050) were used to embed the VOs in resin. The prepared VOs were encased in Spurr resin and hardened at 60 °C for 2 days and left to cool. The Leica Ultracut S UltraMicrotome was used to cut samples to 200 nm which were placed on formvar-coated copper grids. Samples were stained with UA-Zero EM Stain (Agar, AGR1000) for 5 minutes, washed and stained with lead citrate (TAAB, L037) for 5 minutes. The JEOL JEM 1400plus TEM was used for imaging. Images were quantified using ImageJ with the Lam et al^13^, protocol.

### Generating mice ischemic hind limb model, organoid labeling and injection, blood recovery assessment by Laser Doppler imaging, and cell tracking by Bruker fluorescent imaging

To track and compare the effects of human iPSCs-derived DB-VOs against ND-VOs on regeneration, 12 male NOD.CB17 Prkdcscid/NcrCrl mice (10-12 weeks old) were randomly assigned 2 experimental groups of DB-VOs (*n* = 6) or ND-VOs (*n* = 6). Animals were anesthetized with isoflurane (2% at 1 L/minute O*2* for induction, reduced to 1.75%-2% for maintenance; evaluated by sensorimotor reflex) and placed in the supine position on a heating pad with core body temperature maintained at 37 °C prior to administration of presurgical analgesia (10% buprenorphine,150 μL/site s.c.) and eye drops (Viscotears) to prevent corneal dehydration. Hair removal cream was applied to the left leg and inguinal region and cleaned with sterile PBS before further sterilization of the operative area with 70% ethanol and PBS. A small skin incision (~1 cm) was then made in the left inguinal region to facilitate isolation and permanent ligation of the femoral artery to induce experimental hind limb ischemia. Prior to surgery, an equal number of DB-VOs and control ND-VOs were labeled by LuminiCell Tracker 670—Cell Labeling Kit (SCT011, Sigma-Aldrich), 0.8 nm for 12 hours in Stempro34 complete medium, and 6-8 VOs (50 mg in total) per mouse were weighed and disrupted using a 1-mL syringe (21 and 25 gauge). Immediately after femoral artery ligation, small clumps of cells suspended in 200 µl PBS were injected intramuscularly into 3 points of the adductor muscle adjacent to the site of ligation. The skin was then closed with 6/0 Ethilon suture, and mice were allowed to recover on the heated pad (37 °C) and returned to their cage. Limb blood reperfusion was assessed using a Laser Doppler Perfusion System (Moor Instruments, UK) at 0, 1, and 14 days post-surgery and expressed as a ratio of the left (ischemic) to right (nonischemic).

### Dissociation of the VOs for single-cell sequencing

After 14 days, animals were humanly sacrificed following approved guidelines as outlined in “The Humane Killing of Animals under Schedule 1 to the Animals (Scientific Procedures) Act 1986.” Specifically, mice were terminally anesthetized (2% isoflurane at 1 L/minute O_2_) prior to cervical dislocation at vertebrae 1-3 and exsanguination by cutting off the femoral artery to ensure the cessation of blood flow. After sacrifice, tissue from both legs was harvested and subjected to imaging with the In-Vivo Xtreme Imaging System (Bruker, Germany). The biodistribution of labeled injected cells was identified via Bruker Molecular Imaging (BMI) Software, and the area was harvested and embedded into a plastic cylinder containing optimal cutting temperature (OCT) compound and snap-frozen in precooled isopentane (2-methylbutane) within liquid nitrogen. The cylinders (on the dry ice within a Petri dish) were reimaged by the Bruker system to exclude any cylinder without injected cells of VOs and to locate precise regions for subsequent cryosectioning for histological analysis of injected cells and investigation of their integration into host vasculature.

To dissociate the VOs for single-cell analysis, 5-10 VOs were transferred into 2-mL microtubes and washed 3 times with PBS. Then, they were mechanically disrupted by a 21G needle inside a 3 mL DLD dissociation solution (PluriSTEM **D**ispase-II Solution, **L**iberase, #5401119001 **D**Nase I, #10104159001, all from Merck), and incubated for 20-30 minutes at 37 °C. Cell suspensions were subsequently incubated in 50% FBS-containing (Gibco) PBS on ice, filtered through cell strainers of 40 and 30 µm, and centrifuged for 5 minutes at 500 × *g* to get single-cell pellets before staining with flow cytometry antibodies or proceeding to single-cell sequencing experiments.

### Single-cell RNA sequencing (scRNA-Seq) library preparation and sequencing

Twelve DB iPSC-derived VOs (*n* = 3, 4 VOs per line) were evenly pooled together on day 21 of treatment, and the same procedure was performed in parallel for ND controls. They were dissociated within DLD solution and a 1-mL syringe (21 and 25 gauge), neutralized with 50% FBS in PBS, pelleted by centrifugation at 250 × *g* and 4 °C for 5 minutes, washed once with 0.04% BSA in PBS, filtered twice with pluriSelect 40 µm/30 µm cell strainers. The viability of cells was double-checked both with trypan blue staining and automated counter Countess (Life Technology, AMQAX1000) to ensure >90% viability before sequencing. The single cells were diluted to 700-1200 cells/µL with 0.04% BSA in PBS and handed over to the Queen’s Genomics Core Technology Unit (GCTU) for immediate droplet generation (cell capturing) by 10x Genomics Chromium Controller. The Chromium Single-Cell 3ʹ Reagent Kits v2 Chemistry (10x Genomics) was used according to the manufacturer’s instructions. A total of 20 488 cells of dissociated VOs (7311 cells from a mixture of 3 non-diabetics, 13 177 cells from a mixture of 3 diabetics) were captured for Single-cell RNA sequencing (scRNA-Seq) library preparation and paired-end sequencing using the NovaSeq 6000 SP100 (Illumina) carried out at the GCTU of Queen’s University Belfast. Each sample provided ≈4-6 × 10^8^ 150 bp paired-end reads.

### Single-cell RNA sequencing (scRNA-seq) data processing

Reads’ mapping to human reference genome version 38 (GRCh38) and counting were processed using the Cell Ranger software v5.1.2 (10x Genomics). The output files containing the single-cell counts were further analyzed in the Partek Flow software (Partek Inc.) and Bioconductor Packages in R. After the quality control step, the filtered expression matrix was normalized as count per million (CPM). Then, the principal component analysis (PCA) was performed on the counts of log2normalized unique molecular identifiers (UMIs) to reduce the dimensionality of the features/genes. Using the first 20 principal components, t-distributed stochastic neighbor embedding (t-SNE) and uniform manifold approximation and projection (UMAP) were constructed on 2 or 3 dimensions to identify cell clusters. Subsequently, cell types were established based on the expression of their typical biomarkers.

### Differentially expressed genes, cell-cell signaling networks, and gene set enrichment analysis

First, differentially expressed genes (DEGs) for each cluster or group of cells were generated using the PartekFlow gene-specific analysis (GSA) package. To exhibit the relative expression levels of the selected DEGs, Enhanced Volcano plots and Boxplot/violin plots were generated using either PartekFlow or R 4.2.1. Normalized log2UMI expression values were used to produce hierarchical clustering and Heatmaps. Pseudotime analysis was carried out using the Monocle2 package (Version 2.22.0) within PartekFlow. Biomarkers for each cluster or group of cells were computed by PartekFlow, and the list of significant biomarker genes with *P*-value < .05 was used as input for Enrichr to establish cell identities of the clusters.

Additionally, the count matrix and attributes output from PartekFlow were used to create a Seurat object. Seurat Version 4.3.0 functions such as NormalizeData(), ScaleData(), and FindVariableFeatures() were used to normalize the raw data, scale, and find variable features, respectively. Other Seurat functions such as RidgePlot(), VlnPlot(), DotPlot(), and DoHeatmap() were used to generate different plots in R.

CellChat and patchwork were used to generate plots related to cell-cell communications and signaling networks. Secreted signalings were subset from the CellChat database.

Further downstream analysis was done by gene set enrichment analysis (GSEA). Only significant (FDR < 0.05) upregulated and downregulated genes were used for preranked GSEA performed by fgsea (R package) using MSigDB Hallmark dataset. Consequently, significantly positive and negative Hallmarks of the dataset for DB-VOs versus ND-VOs were represented on the bubble plot and GSEA plot.

### Western blot

Cells were harvested and washed with cold PBS, resuspended in RIPA lysis buffer (89900, ThermoFisher) supplemented with protease inhibitors (87785, ThermoFisher), and lysed by ultra-sonication (twice, for 6 seconds; Bradson Sonifier150) to obtain whole-cell proteins. The protein concentration was determined using the Bradford Dye Reagent (Bio-Rad 500-0205). Twenty-five micrograms of whole lysate were applied to SDS-PAGE and transferred to Hybond PVDF membrane (GE Health 15259894), followed by standard Western blot procedure. The membrane was subjected to antibodies of CD31 (ab28364) and GAP43 (PA585949, ThermoFisher), and b-Actin (MAB8929, Bio-techne).

## Results

### Reproducible generation of blood vessel organoids from iPSCs derived from DB and ND donors

**Figure 1.**
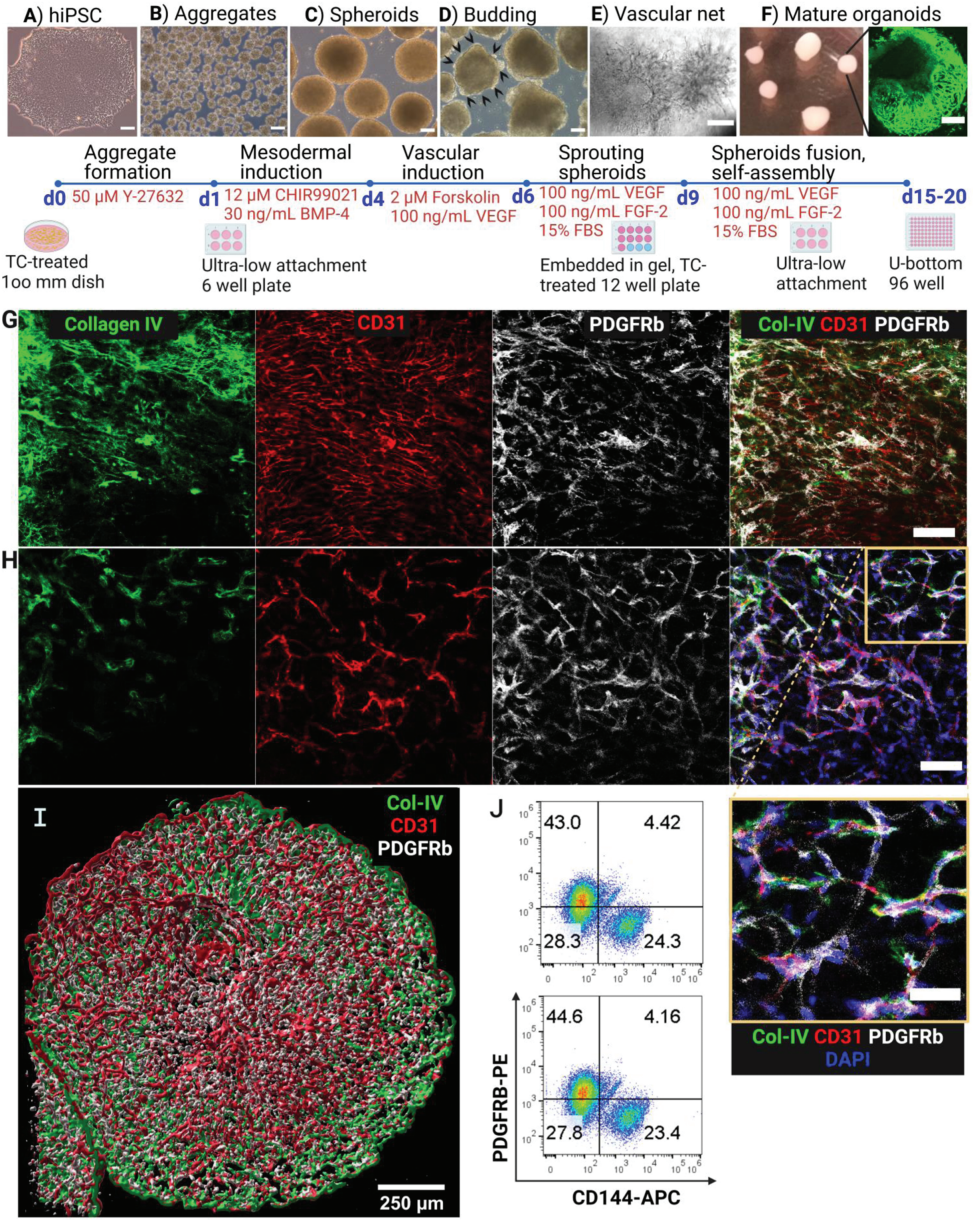
Blood vessel organoid generation and culture from iPSCs. (A) Colony of human iPS cells in 80%-90% confluence were dissociated for cell aggregate generation. (B) Aggregate formation efficiency. The size of aggregates is on average 200 µm. (C) Cell aggregates become bigger to form mesodermal floating spheroids. (D) The floating spheroids start to bud after induction by VEGF-A and forskolin. (E) Phase-contrast image of the sprouting vessels that appeared 2 days after embedding in the 3D Collagen-Matrigel matrix. Every 2-4 vascular spheroids were cut to allow for making full, rounded, mature organoids till day 20 (F). Live staining of the mature organoids by Calcein-AM in green. Scale bars of A-F = 250 µm. The corresponding day, the aim of each stage of differentiation, and the respective materials are indicated below each image (A-F). Successful and reproducible generation of vascular organoids (VOs) from iPS cells that contain both small capillary networks (G) and bigger arteriole (H). Scale bar = 100 µm. Video of the whole confocal series is represented in Supplementary Materials. The confocal imaging showed the presence of both endothelial tubes (CD31^+^, red), mural cells (PDGFRb^+^, gray), and basement membrane (CollagenIV^+^, green) within the VOs (ND5 VO as a representative image). The inset (Scale bar = 50 µm) shows the interaction and alignment of ECs with mural cells. (I) 3D projection of 69 confocal images of a mature VO showing a nice alignment of mural cells with endothelial tubes. Scale bar = 200 µm. Size range of organoids 0.5-1.5 mm. (J) Flow cytometry results indicate the percentage of endothelial cells (CD144+) compared to pericytes (PDGFRB+) in the ND-VO (ND19, upper quadrant) in contrast to the DB-VO (DB14, quadrant).

The efficient and reproducible generation of VOs is essential for their use as a drug testing platform or in investigating underlying cellular and molecular characteristics in vitro. To achieve this, colonies of iPSCs in 80%-95% confluence (Figure 1A) were dissociated into cell clusters to induce the formation of homogeneous cell aggregates of 200 µm in size (Figure 1B). It was critical to not dissociate iPSCs into single cells but into 3-10 cell clusters for efficient aggregate formation (between 1000 and 1500 aggregates from one 6-well plate). The aggregates then underwent directed differentiation toward the mesodermal lineage and grew in the form of floating spheroids (Figure 1C). Next, these floating spheroids were induced to initiate endothelial tube budding from the edge (Figure 1D) to subsequently form vascular networks after embedding in the 3D Collagen I-Matrigel matrix (Figure 1E). Finally, every 3-5 vascular networks in the 3D environment were cut together on day 10 (Supplementary Video S1); allowing them to self-assemble into 3D floating, mature organoids culminating with smooth well-delimitated borders between days 15 and 20 (Figure 1F). The whole process from iPSCs to fully mature organoids is highly reproducible, and it is performed over a 20-day period. At that stage, the VOs (1-1.5 mm in size) were ready for downstream analysis.

To investigate cellular composition, the mature VOs were stained with antibodies against PECAM1 (CD31) and PDGFR-ß, to detect ECs and pericytes, respectively. Confocal images revealed the presence of both cell types (Figure 1G-1I), confirming the successful generation of vascular networks from both DB and ND donors of iPSCs (Supplementary Figure S2). As shown in Supplementary Figure S3A, S3B, the primary plexus formation in VOs is mimicking the embryonic pattern of vascular development.^14^ Interestingly, confocal images in the z-stacks showed the presence of both small network of capillaries (Figure 1G; Supplementary Figure S4A, S4B, Video S2) and larger arterioles as well (Figure 1H; Supplementary Figure S4A, S4B, Video S2), confirming maturation and organization of the organoids. In particular, endothelial tubes in branching points were covered by pericytes as expected.

### Diabetic VOs revealed significantly enhanced ROS production and high Ac-LDL uptake by vascular cells

**Figure 2.**
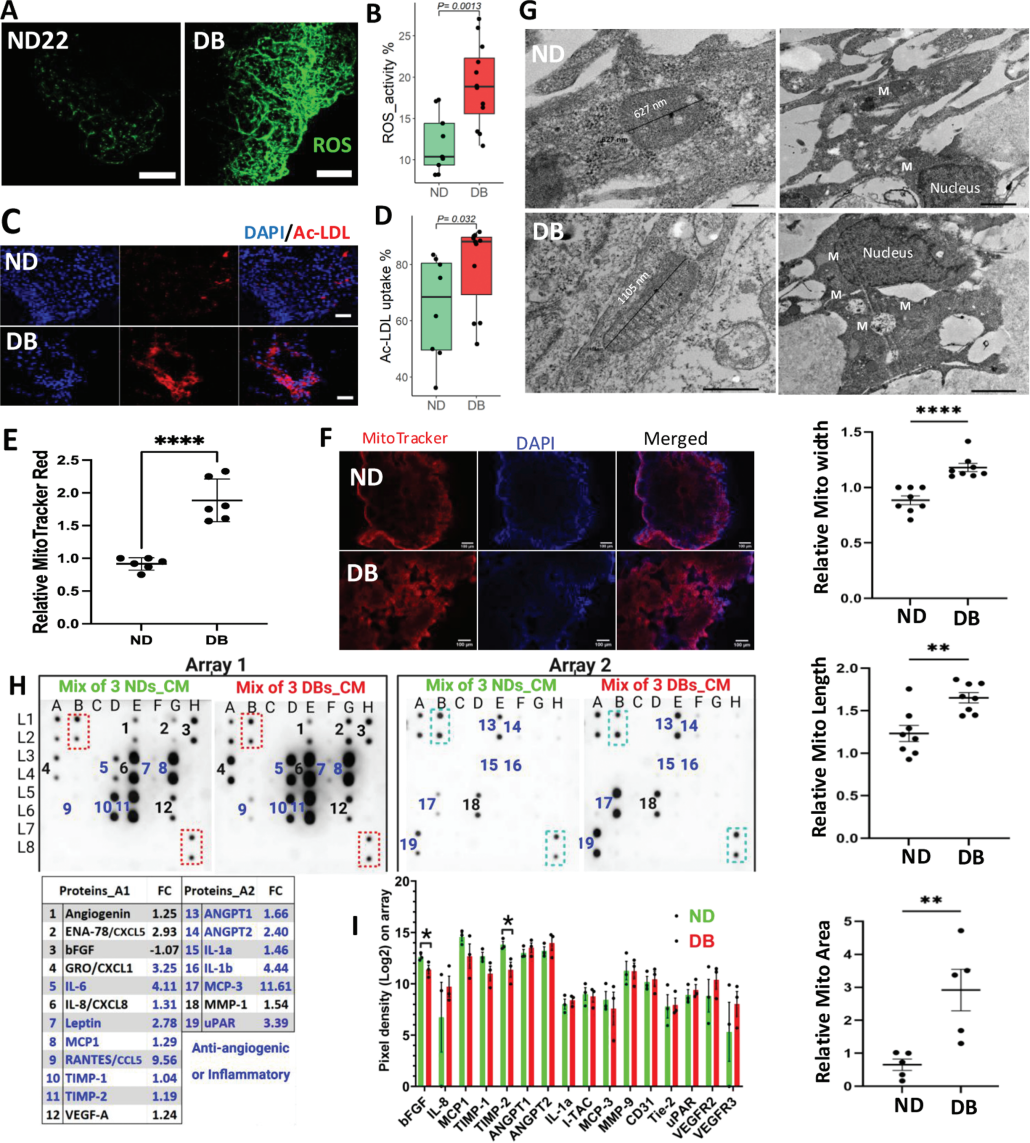
Functional assays of diabetic vascular organoids (DB-VOs) versus non-diabetic (ND-VOs). Enhanced ROS production in DB-VOs is represented by confocal imaging live (A), and a boxplot of flow cytometry analysis of 12 independent experiment of 6 DB-VOs against 9 independent experiment of 5 ND-VOs donors, all listed in Table 1 (B). Ac-LDL uptake showing by confocal imaging (C), and quantified by flow cytometry from 2 independent experiments of 6 DB-VOs (DB07, DB09, DB11, DB12, DB13, and DB14) and 4 ND-VOs (ND5, ND19, ND20, and ND22) represented by boxplot (D), which were significantly higher in DB-VOs as compared to ND-VOs. Scale bar in (A) and (C) is 100 and 200 µm, respectively. (E) Quantification of MitoTracker Red Live Staining of VOs in the bar chart showing significantly higher accumulation of mitochondria in DB-VO as compared to ND-VOs. These data are based on 6 independent experiment of 3 DB-VOs and ND-VOs, *P*-values are shown: ****P < .0001 (unpaired, 2-tailed t-test). (F) Confocal imaging confirms the result of E. (G) Transmission electron microscopy shows larger mitochondria in DB-VOs as compared to ND-VOs. Error bars represent mean ± SEM (*n* = 3). The scale bars for ND are 200 nm and 1 µm, for DB 500 nm and 2 µm, left to right, respectively. *P*-values are shown: **P < .01, ****P < .0001 (unpaired, 2-tailed *t*-test). The human protein array of both conditioned media (minus FBS), 1.5 mL per well for 24 hours, the mixture of 3 DB-VOs (DB07, DB13, and DB14) versus 3 ND-VOs (ND5, ND19, and ND22) 6 weeks post-treatment (H), and 3 independent DB-VOs and ND-VOs individually 3 weeks post-treatment (I). Forty-three angiogenesis-related proteins were compared. Nineteen proteins were up/downregulated in CM’s of DB at day 42 of diabetogenic media treatment, from which 12 of them were antiangiogenic or proinflammatory cytokines (shown in blue text on the arrays’ membrane and quantified in the table). DB-VOs were treated with diabetogenic media (glocuse + TNF-a + IL-6) and control only with mannitol. Left panel: array 1, and right panel: array 2. Values in the table represent the fold change ratio of each protein in DBs versus NDs. Dashed rectangles on the array membranes show the positive internal control for the normalization of each array. Bar plot in (I) showing the comparison of protein extracts from 3 independent DB-VOs versus 3 independent ND-VOs. Only bFGF and TIMP-2 were significantly different between DBs and NDs at 3 weeks post-treatment. A table showing the layout of all 43 antibodies against 20 proteins on array 1, and 23 proteins on array 2, along with the array membrane have shown in Supplementary Figure S6. Unpaired *t*-test nonparametric (Mann-Whitney *U* test) was used to statistically examine differences between DBs and NDs. Mito or M denotes mitochondria. Abbreviation: CM, conditioned media.

Elevated oxidative stress in the vasculature has been linked to the early stages of atherosclerosis,^15^ which can lead to vascular cell dysfunction and activate proatherogenic mechanisms. In diabetes, high glucose and proinflammatory cytokines can dramatically increase ROS levels, resulting in oxidative stress that damages DNA, proteins, and lipids.^7^ Our confocal imaging and flow cytometry analyses revealed significantly higher ROS production in DB-VOs treated with the diabetes-simulating stress (33 mM glucose, 1 ng/mL TNF-a, 1 ng/mL IL-6) for 21 days compared to ND-VOs (Figure 2A, 2B; median ratio = 1.81, *P*-value = .001). Oxidative stress is central to endothelial dysfunction and can be either a cause or a consequence.^16^

Studies have shown that increased intracellular ROS primes vascular cells for the uptake of modified LDL (modLDL), subsequently leading to the formation of smooth muscle cell-derived foam cells.^17^ Elevated uptake of modified LDL, such as Ac-LDL, can elicit further vascular cell dysfunction or activation, initiating and exacerbating atherosclerosis.^18-21^ Our results of confocal imaging and flow cytometry analysis showed that Ac-LDL was avidly taken up by vascular cells in DB-VOs compared to ND-VOs (Figure 2C, 2D; median ratio fold of 1.28, *P*-value = .03). The uptake of modified LDLs is known to cause foam cell formation, but not native LDL alone.^22^

Live staining of VOs showed a significant accumulation of MitoTracker Red in DB-VOs as an index of higher mitochondrial activity (Figure 2E), which was further confirmed by confocal imaging (Figure 2F). The number and size of mitochondria were significantly higher in DB-VOs than in ND-VOs (Figure 2G). Transcriptome-based cellular component analysis of gene ontology confirmed the highly significant enrichment of mitochondrial compartments in DB-VOs versus ND-VOs (Supplementary Figure S5).

### Diabetic VOs revealed significantly higher proinflammatory and antiangiogenic proteins in their secreted media

Inflammation plays a crucial role in the pathogenesis of atherosclerosis,^23,24^ and vascular cells have been implicated in this process.^23^ In addition, transcriptional memory can lead to faster and greater signal-dependent transcription of disease-related genes.^25,26^ To simulate diabetes-induced low-grade inflammation, we exposed DB-patient iPSCs-derived VOs to high glucose (33 mM) and low concentrations of proinflammatory cytokines (1 ng/mL of TNF-a and IL-6) to induce pathological pathways. Using a human antibodies array, we compared secreted proteins in the conditioned media of ND-VOs and DB-VOs on day 42 of treatment (Figure 2H; Supplementary Figure S6A) as well as the protein extracts of VOs on day 21 (Figure 2I; Supplementary Figure S6B). Our results revealed higher levels of antiangiogenic proteins and inflammatory cytokines in the secreted media of DB-VOs compared to ND-VOs (Figure 2H). Specifically, RANTES, IL1b, and MCP3 had fold changes of 9.56, 4.4, and 11.61, respectively, in the secreted media of DB-VOs compared to ND-VOs. However, no significant difference was observed between the protein extracts of DB-VOs and ND-VOs on day 21 of treatment (Figure 2I).

### Diabetic VOs represent less blood perfusion recovery and poorer integration into host vasculature than ND-VOs

**Figure 3.**
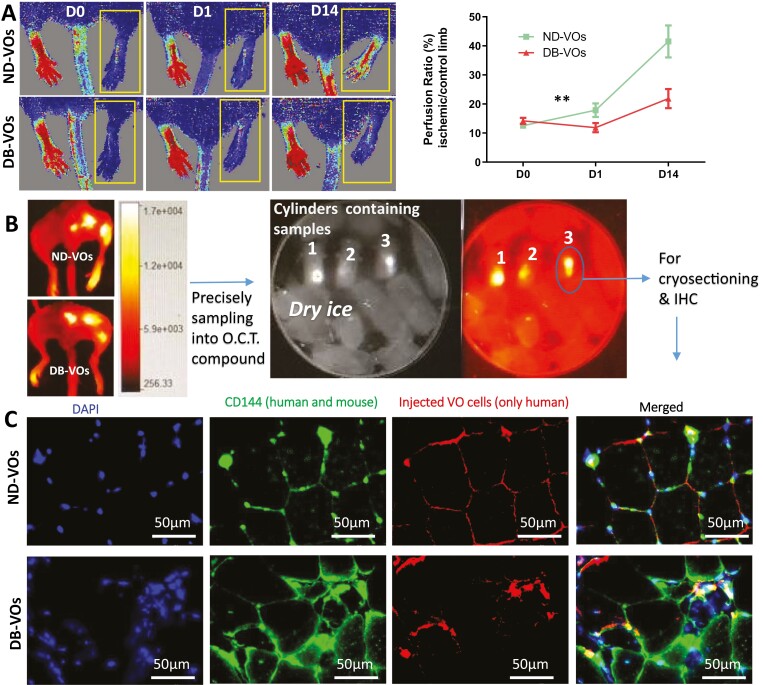
Comparison of diabetic (DB-VOs) and non-diabetic vascular organoids (ND-VOs) in hind limb ischemia recovery and host vasculature integration. (A) Laser Doppler imaging was used to assess blood reperfusion in the lower limb of SCID mice at days 0, 1, and 14 post-surgery following transplantation of DB-VOs (DB07, DB9, and DB14) and ND-VOs (ND5, ND19, and ND22). The yellow rectangle highlights the ischemic leg, with the blood perfusion recovery quantified in the line chart (*n* = 6 per condition, 2-way ANOVA, *P*-value < .01). The average blood perfusion recovery was 41.5% for ND-VOs and 21.8% for DB-VOs. (B) Injected labeled cells were tracked at 3 locations around the hind limb ischemia area by Bruker fluorescent imaging on day 14 after sacrificing mice. The muscles were sampled precisely from the injected area and embedded into Tissue-Tek O.C.T. compound within cylinders. The cylinders were then tracked for labeled human cells by Bruker imaging on dry ice to avoid heat damage to snap-frozen tissue samples. (C) Cryosectioning of the cylinders containing injected cells of labeled VOs was performed, followed by IHC and confocal imaging to assess the integration of injected human cells (red, labeled with LuminiCell Tracker 670) of ND-VOs and DB-VOs into host vasculature. The results indicated that injected cells of ND-VOs were better integrated with host vasculature than those of DB-VOs.

To compare the effects of DB-VOs and ND-VOs on regeneration, we injected equal numbers of labeled DB-VOs and ND-VOs into the ischemic hind limb muscle of male NOD.CB17 Prkdcscid/NcrCrl mice (*n* = 6 per condition) following femoral artery ligation. The blood perfusion recovery was assessed using Laser Doppler imaging system at 0, 1, and 14 days post-surgery. We found that ND-VOs showed significantly better blood perfusion recovery than DB-VOs (41.5% vs 21.8%, *P*-value < .01; Figure 3A).

To investigate the integration of injected human cells into host vasculature, we harvested and imaged the tissue of both legs with the In-Vivo Xtreme Imaging System on day 14 after the surgery. The biodistribution of labeled injected cells was identified via BMI Software, and the area was harvested and embedded into a plastic cylinder containing OCT compound and snap-frozen in precooled isopentane within liquid nitrogen. We tracked the injected labeled cells at 3 locations around the Hind Limb Ischemia area by Bruker fluorescent imaging. The muscles were precisely sampled from the injected area and embedded into Tissue-Tek O.C.T. compound within cylinders. The cylinders were then tracked for labeled human cells by Bruker imaging on dry ice to avoid heat damage to snap-frozen tissue samples (Figure 3B). Cryosectioning of the cylinders containing injected cells of labeled VOs was performed, followed by IHC and confocal imaging to assess the integration of injected human cells of ND-VOs and DB-VOs into host vasculature. The results indicated that injected cells of ND-VOs were better integrated with host vasculature than those of DB-VOs (Figure 3C).

### Characterization of the iPSC-derived VOs in single-cell resolution

To assess the cell composition and functions of ND-VOs and DB-VOs, we performed single-cell transcriptome profiling on day 21 of treatment using organoids from 3 independent iPSC lines for each group of DBs and NDs. A total of 7311 cells and 13 177 cells were captured in the ND-VO and DB-VO samples, respectively (Supplementary Figure S7). In the quality control step, we excluded cells with <500 detected genes, which were considered debris or low-quality cells, as well as cells with >7000 detected genes, which were potentially doublets or multiplets (Supplementary Figure S7). We also filtered out cells with low (<0.5%) or high (>50%) levels of mitochondria-encoded genes. As a result, a total of 3782 cells out of 20 488 were excluded, leaving 16 706 VOs’ cells (5799 NDs and 10 907 DBs sample) with ≈ 500-60 000 unique molecular identifier counts/cells for downstream analysis.

**Figure 4.**
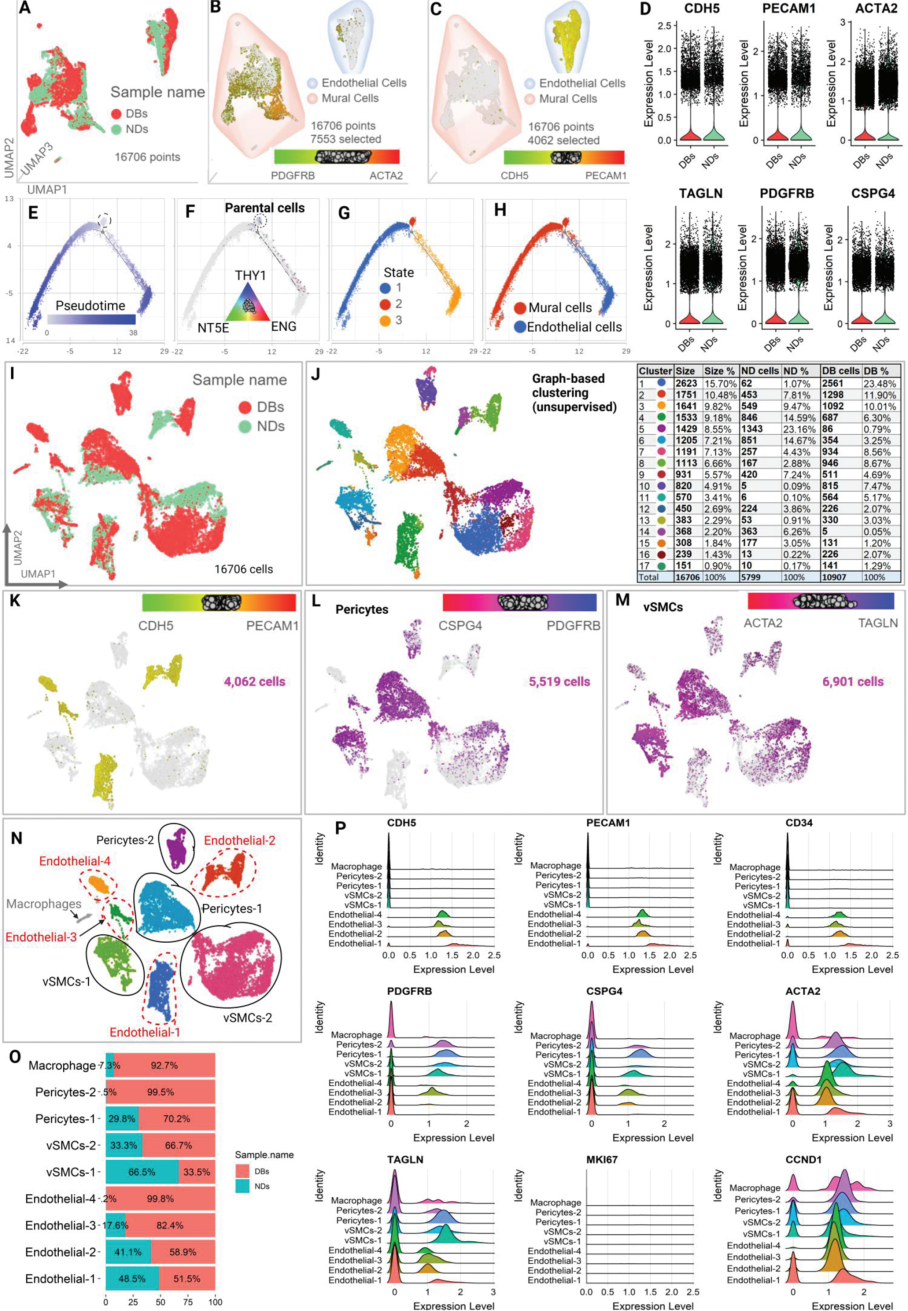
Characterization of the iPSC-derived vascular organoids (VOs) in single-cell resolution. (A) 3D UMAP projection of the cells’ pool of VOs derived from diabetic (DB07, DB09, and DB11) and non-DBs (ND05, ND19, and ND22) donors, coloured by the sample name. (B) Mural cells’ subpopulation showing the expression of PDGFRb and ACTA2 (a-SMA). (C) Endothelial cells’ subpopulations showing the expression of PECAM1 (CD31) and CDH5 (CD144). (D) Differentially expressed analysis of total single cells of DB-VOs versus ND-VOs showed no significant differences in the expression of typical markers of endothelial cells (CDH5 and PECAM1), vSMCs (TAGLN), and pericytes (PDGFRB and CSPG4), represented by violin plots, except for vSMCs’ marker ACTA2 with FDR < 0.009 and fold change = −2.2 (See Supplementary Excel Sheet). The expression level is normalized log2-UMI and each dot represents a cell. (E, F) Pseudo time analysis shows a common ancestor cell expressing mesenchymal stem cell markers (CD44, CD73/NT5E, CD90/THY1, and CD105/ENG), further developed into the more differentiated cell during the time (darker blue). (G) VOs represent 3 cell states with a branch point, reflecting 2 differentiated cell populations with a common progenitor cell. (H) Overlying cell identities found in (B) and (C) on the trajectory revealed that the dichotomy is mural cells in the left wing and ECs in the right wing. (I) 2D UMAP projection of the VOs’ single cells coloured by the sample name. (J) Seventeen graph-based unsupervised clusters with their respective cell number and percentage in DB-VOs and ND-VOs in the table. (K) Endothelial cells (ECs) were distinguished into 4 clusters based on the expression of PECAM1 and CDH5. (L) Pericytes were identified based on the expression of CSPG4 (NG2) and PDGFRB. (M) vSMCs were characterized by the expression of ACTA2 (a-SMA) and TAGLN (SM22). (N) Classification of the whole 16 706 cells into a total of 9 clusters based on the distinct clusters of ECs, VSMCs, and pericytes. A small population of macrophages was identified based on the expression of CD14 and CD86 (data not shown). (O) The percentage of each vascular cell subtype within DB-VOs and ND-VOs. Endothelial-4 and pericytes-2 are specific for DB-VOs. (P) The relative expression of some selected markers in each of the 9 main subpopulations.

Single-cell transcriptomic analysis of ND-VOs and DB-VOs on 3-dimensional UMAP revealed the presence of 2 distinct clusters of cells, which were identified as mural cells and ECs based on their expression of pan-specific markers (Figure 4B, 4C). There were no significant differences in the expression of typical markers of endothelial and mural cells between the 2 groups of VOs, indicating that the differentiation protocol was reproducible (Figure 4D). However, the vSMCs’ marker ACTA2 was downregulated in DB-VOs compared to ND-VOs, suggesting some differences in the vSMCs population. Further investigation of the data using pseudotime and trajectory analysis revealed a common parental cell that expressed mesenchymal stem cell markers (CD44, CD73/NT5E, CD90/THY1, and CD105/ENG). This parental cell gave rise to 2 differentiated cell populations that were located at a branch point on the trajectory (Figure 4G). One of these populations differentiated into mural cells (cell state 1) located in the left wing of the trajectory, while the other population differentiated into ECs (cell state 3) located in the right wing of the trajectory (Figure 4H). These data provide evidence for the reproducible differentiation of iPSCs toward VOs, with the generation of 2 distinct cell populations that closely resemble mural cells and ECs.

Dimensionality reduction was performed using PCA and the first 20 components were subjected to Graph-based unsupervised clustering and UMAP projection in 2 dimensions, resulting in 17 clusters (Figure 4I, 4J; Supplementary Excel Sheet S1) in pooled DB-VOs and ND-VOs, to interrogate DB-induced vascular cells’ transcriptome differences. The number of cells in each cluster varies, ranging from 151 to 2623 cells per cluster (Figure 4J, table). Differentially expressed genes in DB-VO versus ND-VOs in each cluster are represented in Supplementary Excel Sheet S2. Each cluster possesses a unique set of DEGs against the rest of the cells^27^ that were also used as biomarkers to annotate each cluster into a specific cell type (Supplementary Excel Sheets S3 and S4).

The distinct clusters of vascular cells were identified based on the expression of typical biomarkers. ECs were distinguished into 4 clusters based on the expression of PECAM1 and CDH5 (Figure 4K). Pericytes were identified based on the expression of CSPG4 (NG2) and PDGFRB (Figure 4L), while vSMCs were characterized by the expression of ACTA2 (a-SMA) and TAGLN (SM22; Figure 4M). Based on the distinct clusters of ECs, VSMCs, and pericytes, a total of 9 populations were defined (Figure 4N). Endothelial-4 (EC4) and pericytes-2 populations were specific to DB-VOs (Figure 4O). The expression of CD32, CD144, and CD34 were highly specific to endothelial populations, while PDGFRb, CSPG4, ACTA2, and TAGLN represented a bright expression in mural cells and dim expression in ECs (Figure 4P). The biomarkers of the 9 cell populations are detailed in Supplementary Excel Sheet S5, with the top biomarkers shown in Supplementary Figure S8. Additionally, differential gene expression analysis of each population in DB-VOs versus ND-VOs is represented in Supplementary Excel Sheet S6.

**Figure 5.**
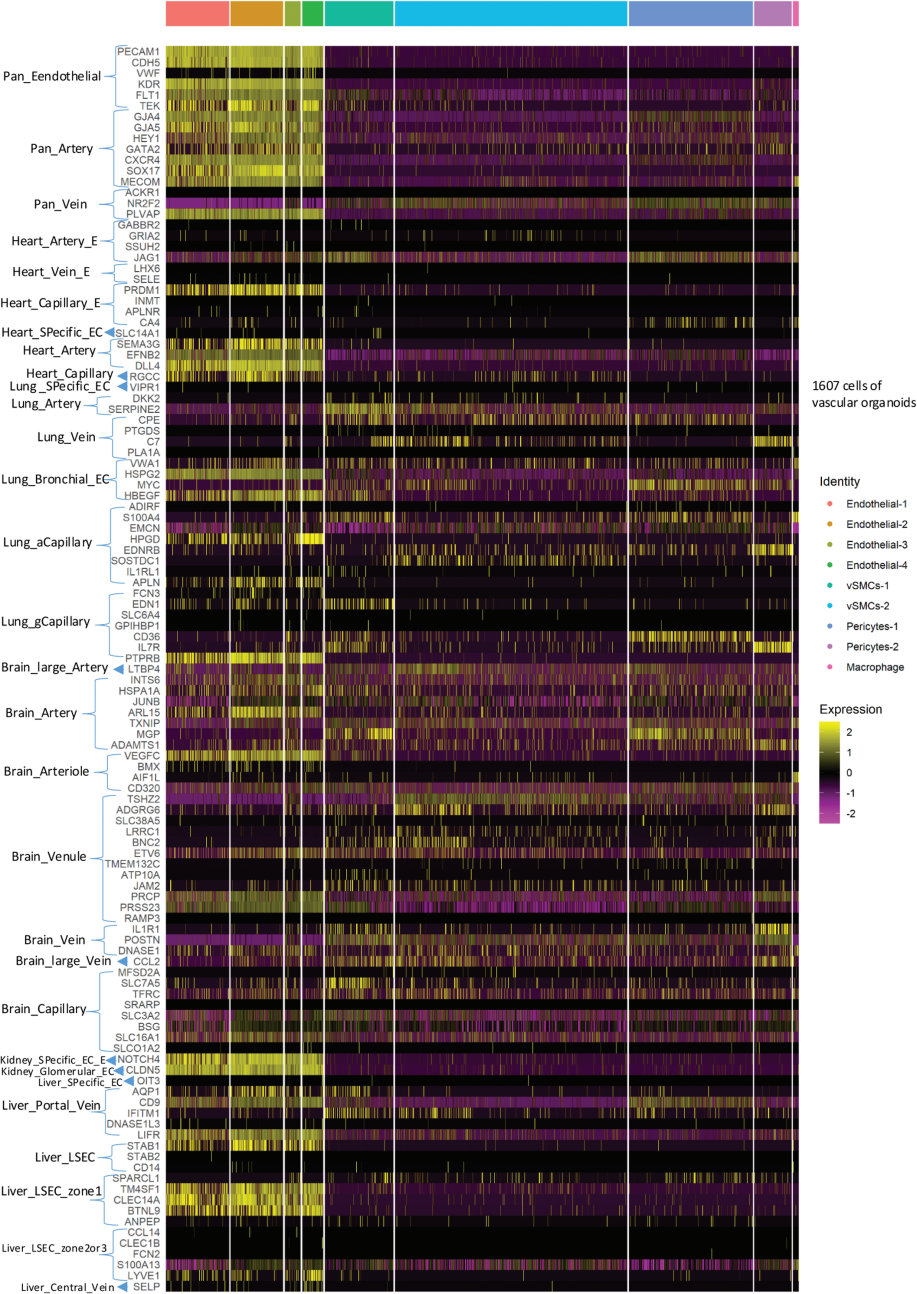
Lack of full similarity of EC populations in vascular organoids (VOs) to organ-specific EC subtypes. The heatmap shows the degree of similarity between ECs in VOs and organ-specific subtypes of ECs found in the heart, brain, lung, kidney, and liver. Biomarkers for each organ were obtained from Table 3 of a recent publication (Trimm et al, 2023, Nature). The heatmap reveals that the endothelial 1-4 in VOs only partially express a few markers from each organ, indicating that they do not match any specific organ perfectly. These results highlight the need for developing organ-specific differentiation protocols for iPSCs to effectively produce VOs that closely resemble a desired organ. Abbreviations: aCap, aerocyte capillary; EC, endothelial cell; E, embryonic; gCap, general capillary; LSEC, liver sinusoidal endothelial cell.

The biomarkers of organ-specific subtypes of ECs found in the heart, brain, lung, kidney, and liver were obtained from Table 3 of a recent publication^28^ to investigate whether each of the 4 clusters of ECs corresponds to either artery, vein, or capillary. The results (Figure 5) showed that the VOs corresponded mainly to pan-artery markers rather than vein or capillary. However, the ECs in VOs only partially expressed a few markers from each organ, suggesting that they do not match any specific organ perfectly. This highlights the need for developing organ-specific differentiation protocols for iPSCs to effectively produce VOs that closely resemble a desired organ. Furthermore, different lists of typical markers for vascular cells were also tested but failed to establish each of the 4 clusters of ECs as a clear-cut artery, capillary, vein, lymphatic, or tip cell identities (data not shown).

**Figure 6.**
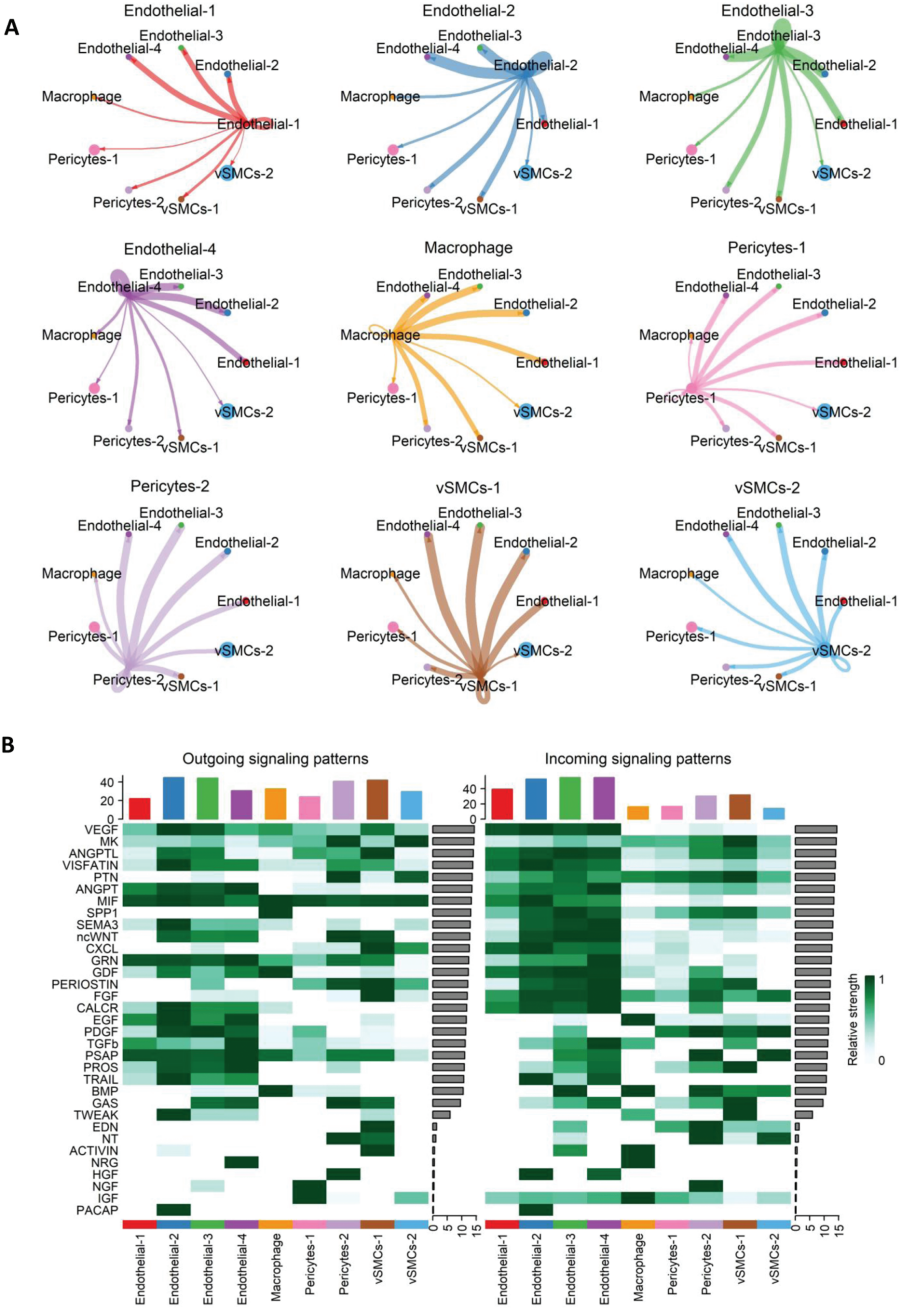
Cell-cell signaling network in diabetic vascular organoids (DB-VOs). (A) Analysis of the signaling sent from each cell group using the CellChat algorithm (R package). Nodes represent cell groups, and edges indicate signaling interactions, with thickness reflecting signaling strength. (B) The heatmap represents 33 significantly secreted signaling molecules in the cell-cell communication network. This highlights which signals contribute the most to the outgoing or incoming signaling of certain cell groups, providing a comprehensive view of the signaling roles played by various molecules.

We used the CellChat algorithm and database to perform secreted signaling network analysis and investigate how different cell populations within DB-VOs communicate. The analysis showed a complex cell-cell communication network, with nodes representing cell groups and edges indicating signaling interactions of varying strengths. The results revealed that ECs in DB-VOs send stronger signals to other ECs, while mural cells send stronger signals to ECs rather than themselves (Figure 6A). Similar results were obtained for NDs iPSC-derived VOs (Supplementary Figure S9A). The heatmap in Figure 6B identified 33 significantly secreted signaling molecules within DB-VOs, highlighting the signals that contribute the most to the outgoing or incoming signaling of certain cell groups. These results provide a comprehensive view of the signaling roles played by various molecules and help to understand the complex mechanisms of cell-cell communication in VOs.

**Figure 7.**
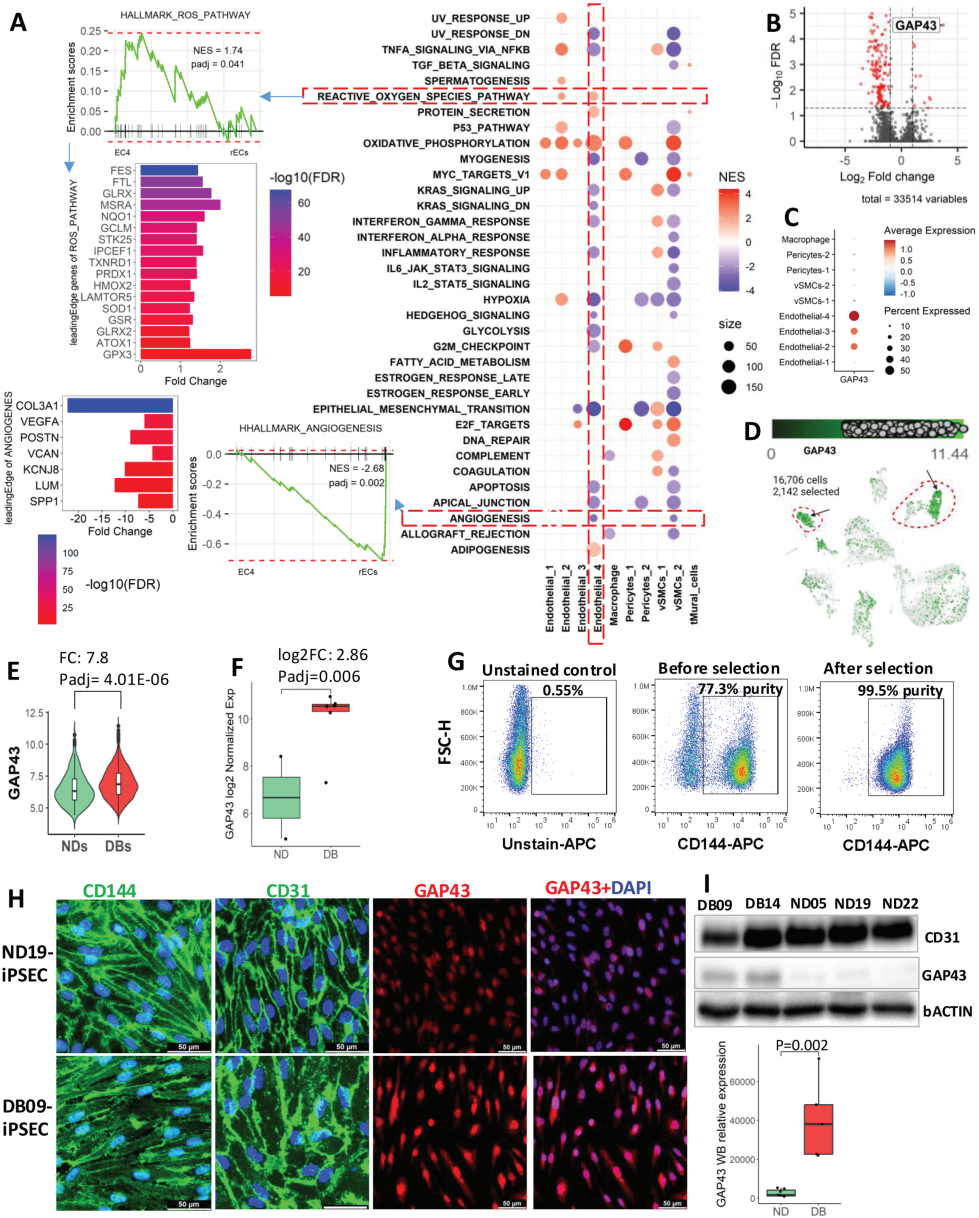
Differentially expressed hallmarks and genes in diabetic vascular organoids (DB-VOs). (A) Bubble plot showing the 35 hallmarks (*y*-axis) that were either overrepresented or underrepresented in at least one of the conditions (*x*-axis). Differentially expressed genes (DEGs) were identified by comparing the 9 main subpopulations/clusters of DBs against NDs, and significant upregulated and downregulated genes were used for preranked gene set enrichment analysis (GSEA) performed by fgsea (R package) and using MSigDB Hallmark dataset. Diabetic-specific clusters, endothelial-4 (EC4) and pericytes-2, were compared versus the rest of ECs (rECs) and rest of the mural cells, respectively. Three additional conditions were included in the analysis: total (t) mural cells of DB vs ND, total ECs of DB vs ND, and total DB cells vs ND cells. However, no significant hallmarks were identified in the latter 2 comparisons, and therefore they are not shown in the bubble plot. Circle size indicates the number of genes in each hallmark dataset. The GSEA plot on the left shows the normalized enrichment score and adjusted *P*-value (padj) for the ROS pathway and angiogenesis hallmarks, which were significantly overrepresented and underrepresented, respectively, in the diabetic-specific EC4 cluster. Bar plots show the leading edge genes’ expression in the DEGs dataset. Pericytes-2 showed no positively enriched hallmarks. (B) GAP43 was identified as the top DEG in the comparison of total ECs of DB versus total ECs of ND, with a fold change of 13.2 and FDR of 2.88 × 10^−5^. (C) GAP43 expression was highest in the diabetic-specific EC4 cluster. (D) GAP43 expression overlay on the uniform manifold approximation and projection (UMAP) plot, with the arrow indicating the DB-specific cell clusters. (E) Violin plot showing that GAP43 has 7.8 times significantly higher expression in total single cells of DB-VOs compared to ND-VOs in scRNA-seq data. (F) Box plot showing the log2 normalized expression value of GAP43 in an independent bulk RNA-seq dataset of pure DB iPSC-derived ECs (DB iPS-EC, *n* = 6, DB03, DB06, DB07, DB09, DB11, and DB23) versus ND iPS-EC (*n* = 2, ND19 and ND22), further confirming the scRNA-seq results. (G) Flow cytometry demonstrating the purity of iPS-EC using CD144 antibody before and after selection of endothelial cells by autoMACS for subculture and analysis of GAP43 expression by immunostaining and Western blotting. (H) Immunostaining of DB iPS-EC with typical EC markers CD144 and CD31, showing higher expression of GAP43. (I) Western blotting to quantify the relative expression of GAP43 in DB iPS-ECs versus ND iPS-ECs, with the box plot representing the normalized signal intensity values of GAP43 in Western blot bands of DB iPS-ECs (*n* = 5) versus ND iPS-ECs (*n* = 8).

To better understand the functional implications of transcriptomic changes in the DB-VOs population compared to ND-VOs, we conducted GSEA (Hallmark) based on DEGs (Supplementary Excel Sheet S6) for each of the 9 populations. The results of this analysis were visualized using a bubble plot of the overrepresented and underrepresented hallmarks in the DB-VOs compared to ND-VOs, as shown in Figure 7A. Intriguingly, we found that the ROS pathway hallmark was significantly enriched in the DB-specific EC4 cluster. This finding supports the notion that oxidative stress and endothelial dysfunction are linked to diabetes. However, when we compared the total ECs and total vascular cells of DB versus ND, we observed no significant overrepresented or underrepresented hallmarks, highlighting the importance of studying subpopulations of cells rather than bulk cell populations. Furthermore, significantly lower angiogenesis hallmark suggests that the transcriptomic changes in the EC4 population may contribute to the dysfunction of a subset of ECs, which in turn may lead to impaired blood vessel function. Further analysis of the DEG dataset’s leading edge genes showed that the ROS pathway hallmark was driven by the upregulation of several genes involved in oxidative stress, including methionine sulfoxide reductase A (MSRA) and glutathione peroxidase 3 (GPX3). These genes are considered antioxidative, playing a crucial role in reducing oxidative stress and protecting cells from damage caused by ROS.

### GAP43 is highly expressed in DB ECs

We conducted further analysis to investigate the top DEGs inDB-VOs compared to ND-VOs. Our results revealed that GAP43 was the top DEG, exhibiting a fold change of 13.2 and FDR of 2.88 × 10^−5^ in the total ECs of DB-VOs compared to ND-VOs (Figure 7B). Next, we examined the expression of GAP43 in each of all the 9 populations in DB-VOs and found that it was predominantly expressed in the DB-specific EC4 cluster and a subset of the EC3 population (Figure 7C, 7D). We further confirmed the elevated expression of GAP43 in DB-VOs by analyzing the scRNA-seq data of total single cells of DB-VOs versus ND-VOs using a violin plot, which showed that GAP43 had 7.8 times higher expression in DB-VOs (Figure 7E). This finding was significant because we were able to investigate GAP43 without sorting for the EC4 population, which can be technically challenging and time consuming. It also has clinical implications, as a diagnostic or therapeutic candidate that does not require sorting would be more valuable.

To validate the scRNA-seq results, we analyzed an independent bulk RNA-seq dataset of pure DB iPSC-derived ECs (iPS-EC; *n* = 6) versus ND iPS-EC (*n* = 2) and found significantly higher normalized log2 expression value of GAP43 in DB iPS-EC (Figure 7F; Supplementary Excel Sheet S7). We also purified ECs from both DB-VOs and ND-VOs using CD144 antibody (Figure 7G) to further examine them by immunostaining and Western blotting. The cells expressed typical EC markers (CD144 and CD31) in culture, and those derived from DB-VOs showed significantly higher expression of GAP43 (Figure 7H), which was further confirmed by Western Blot (WB) quantification (Figure 7I).

## Discussion

ECs perform an essential role in vascular function; however, their interaction with mural cells cannot be overlooked in how they respond to pathological conditions. Our results of confocal imaging and single-cell data revealed that iPSC-derived VOs represent the benefit of having mural cells in addition to ECs. Unlike previous protocols that were only 2D differentiation of ECs from iPSCs,^29^ our 3D differentiation protocol exhibited standard endothelial morphology covered by mural cells. We also showed subpopulations of each cell type and provided a comprehensive view of cell-cell signaling networks within both DB-VOs and ND-VOs.

We were able to reproducibly grow VOs within 15-20 days from iPSCs of both DB and ND donors, with an efficient differentiation protocol. The methodology described in this study for generating VOs from iPSCs is consistent with previous reports.^12^ However, this study further demonstrates the reproducibility of the method for generating VOs from both DB and ND donors. The study also highlights the importance of inducing the formation of homogeneous cell aggregates of a specific size and not dissociating iPSCs into single cells for efficient aggregate formation. Additionally, we provide a detailed protocol for cutting and transferring vascular networks to reduce labor time and risk of contamination, which can be useful for researchers seeking to replicate the method.

Although there were no significant differences in the expression of typical cell markers, we found that DB-VOs had impaired mitochondrial function evidenced by changes in the number, size, and ROS production as compared to ND-VOs. In-depth single-cell analysis revealed that this impaired function is specific to the EC4 population, the DB-specific cluster, evidenced by a significantly higher normalized enrichment score for the ROS pathway (Hallmark) and mitochondrial cellular components (GO-CC), as well as less angiogenesis hallmark. Additionally, as compared to control ND-VOs, DB-VOs showed significantly more modLDL uptake and higher proinflammatory cytokines. These 3 early events (ie, oxidative stress, modLDL uptake, and inflammatory cytokines) work in a vicious cycle in a way that one can worsen the other.^15-17,23^

Moreover, we investigated the organ-specific subtypes of ECs found in various organs^28^ and found that the VOs correspond mainly to pan-arterial markers rather than vein or capillary. This finding is in agreement with a previous study^30^ that showed arterial endothelial genes were highly expressed in iPSCs-ECs, whereas venous endothelial genes had almost no expression. However, the ECs in VOs only partially expressed a few markers from each organ, suggesting that they do not match perfectly to any specific organ. This highlights the need for developing organ-specific differentiation protocols for iPSCs to effectively produce VOs that closely resemble a desired organ.

Previous research by Wimmer et al^9^ demonstrated that human blood vessel organoids could serve as a model for DB vasculopathy. They observed the formation of a stable, perfused vascular tree following transplantation into mice, including arteries, arterioles, and venules. However, our scRNA-seq results indicated a predominant expression of arterial and capillary markers within our VOs, rather than vein markers. It is worth noting that the in vivo implantation by Wimmer et al might lead to better development of the VOs, potentially influenced by environmental signals post-implantation. A recent in vitro study has shown that venous development occurs following arterial development.^31^

In this study, we used single-cell RNA sequencing to investigate diabetes-specific characteristics and intra-organoid differences within iPSC-derived VOs at the single-cell level. The disparities observed between DB-VOs and ND-VOs can be attributed to a complex interplay of factors. These include the inherent epigenetic memory carried by DB-VOs, as well as the impact of the 21-day stimulation with TNF and IL-6, combined with the elevated glucose concentrations (33 mM). Our study intentionally exposed iPSC-derived VOs from patients with diabetes to hyperglycemia and inflammatory cytokines to simulate the epigenetic memory of cells previously exposed to such conditions. The reason for using different culture media for DB-VOs and ND-VOs is to enhance this phenotype in a well-controlled system. By modifying the culture conditions for DB organoids to include factors such as proinflammatory cytokines and elevated glucose levels, we aim to intensify and accentuate the DB phenotype. In contrast, maintaining ND organoids in standard culture conditions serves as a comparative control, allowing us to discern the specific effects of diabetes on vascular function and phenotype. This approach enhances our ability to investigate disease-specific mechanisms within a tightly controlled experimental framework, thereby advancing our understanding of DB vascular pathology. Even when cultured in normal media, the DB-VOs display phenotypic characteristics reflective of the disease (data not shown). In fact, GAP43 is highly appressed in DB iPS-ECs in comparison to ND iPS-ECs when both of them were generated and cultured in normal culture media in the absence of high glucose and cytolines.

We identified GAP43 (Neuromodulin) as a top molecular marker of DB ECs including EC4 and experimentally confirmed its significantly higher expression in the purified ECs population. GAP43 protein is mainly expressed in the brain as a growth-associated protein to assist filopodial extension and branching of neurons^32^ and GAP43 expression can be modulated by diabetes.^33^ Researchers have shown that GAP43 might be a marker of incipient DB neuropathy.^34^ This study highlights GAP43 as a potential molecular marker of DB ECs, which is mainly known for its role in nerve regeneration and plasticity, and its modulated expression by diabetes suggests a role in the development and progression of diabetes-related complications. GAP43 expression in ECs has not been widely reported in the literature, and the increased expression of GAP43 in DB ECs may suggest a role in endothelial dysfunction, a hallmark of DB vascular complications.

Overall, these findings can inform the development of new therapies for DB vascular diseases and contribute to ongoing efforts to develop VOs for disease modeling and drug discovery. This study underscores the need for further studies to explore GAP43 potential as a therapeutic target.

## Supplementary Material

Supplementary material is available at *Stem Cells* online.

sxae043_suppl_Supplementary_Materials

## Data Availability

All relevant data are within the paper and its Supporting Information files. The single-cell RNA sequencing data supporting the findings of this study is available from the corresponding author on reasonable request.

## References

[1] Taylor KS , Heneghan CJ, Farmer AJ, et al. All-cause and cardiovascular mortality in middle-aged people with type 2 diabetes compared with people without diabetes in a large U.K. primary care database. Diabetes Care. 2013;36(8):2366-2371. 10.2337/dc12-1513 23435157 PMC3714501

[2] Domingueti CP , Dusse LMS, Carvalho M das G, et al. Diabetes mellitus: the linkage between oxidative stress, inflammation, hypercoagulability and vascular complications. J Diabetes Complications. 2016;30(4):738-745. 26781070 10.1016/j.jdiacomp.2015.12.018

[3] Giglio RV , Stoian AP, Haluzik M, et al. Novel molecular markers of cardiovascular disease risk in type 2 diabetes mellitus. Biochim Biophys Acta Mol Basis Dis. 2021;1867(8):166148. 10.1016/j.bbadis.2021.166148 33892081

[4] Li M , Qian M, Kyler K, Xu J. Endothelial-vascular smooth muscle cells interactions in atherosclerosis. Front Cardiovas Med. 2018;5(23):151. 10.3389/fcvm.2018.00151 PMC6207093 30406116

[5] Vilà González M , Eleftheriadou M, Kelaini S, et al. Endothelial cells derived from patients with diabetic macular edema recapitulate clinical evaluations of Anti-VEGF responsiveness through the neuronal pentraxin 2 pathway. Diabetes. 2020;69(10):2170-2185. 10.2337/db19-1068 32796081

[6] Yu XY , Geng YJ, Liang JL, et al. High levels of glucose induce “metabolic memory” in cardiomyocyte via epigenetic histone H3 lysine 9 methylation. Mol Biol Rep. 2012;39(9):8891-8898. 10.1007/s11033-012-1756-z 22707199

[7] Luc K , Schramm-Luc A, Guzik TJ, Mikolajczyk TP. Oxidative stress and inflammatory markers in prediabetes and diabetes. J Physiol Pharmacol. 2019;70(6):809-824. 10.26402/jpp.2019.6.01 32084643

[8] Su L , Kong X, Loo SJ, et al. Diabetic endothelial cells differentiated from patient iPSCs show dysregulated glycine homeostasis and senescence associated phenotypes. Front Cell Dev Biol. 2021;31(9):667252. 10.3389/fcell.2021.667252 PMC8201091 34136485

[9] Wimmer RA , Leopoldi A, Aichinger M, et al. Human blood vessel organoids as a model of diabetic vasculopathy. Nature. 2019;565(7740):505-510. 10.1038/s41586-018-0858-8 30651639 PMC7116578

[10] Vilà-González M , Kelaini S, Magee C, et al. Enhanced function of induced pluripotent stem cell-derived endothelial cells through ESM1 signaling. Stem Cells. 2019;37(2):226-239. 10.1002/stem.2936 30372556 PMC6392130

[11] Yang C , Eleftheriadou M, Kelaini S, et al. Targeting QKI-7 in vivo restores endothelial cell function in diabetes. Nat Commun. 2020;11(1):3812. 10.1038/s41467-020-17468-y 32732889 PMC7393072

[12] Wimmer RA , Leopoldi A, Aichinger M, Kerjaschki D, Penninger JM. Generation of blood vessel organoids from human pluripotent stem cells. Nat Protoc. 2019;14(11):3082-3100. 10.1038/s41596-019-0213-z 31554955

[13] Lam J , Katti P, Biete M, et al. A universal approach to analyzing transmission electron microscopy with ImageJ. Cells. 2021;10(9):2177. 10.3390/cells10092177 34571826 PMC8465115

[14] Potente M , Mäkinen T. Vascular heterogeneity and specialization in development and disease. Nat Rev Mol Cell Biol. 2017;18(8):477-494. 10.1038/nrm.2017.36 28537573

[15] Marchio P , Guerra-Ojeda S, Vila JM, et al. Targeting early atherosclerosis: a focus on oxidative stress and inflammation. Oxid Med Cell Longev. 2019;2019(1):8563845. 10.1155/2019/8563845 31354915 PMC6636482

[16] Odegaard AO , Jacobs DRJ, Sanchez OA, et al. Oxidative stress, inflammation, endothelial dysfunction and incidence of type 2 diabetes. Cardiovasc Diabetol. 2016;15(24):51. 10.1186/s12933-016-0369-6 27013319 PMC4806507

[17] Chellan B , Reardon CA, Getz GS, Hofmann Bowman MA. Enzymatically modified low-density lipoprotein promotes foam cell formation in smooth muscle cells via macropinocytosis and enhances receptor-mediated uptake of oxidized low-density lipoprotein. Arterioscler Thromb Vasc Biol. 2016;36(6):1101-1113. 10.1161/ATVBAHA.116.307306 27079883 PMC4882225

[18] Xu L , Wang YR, Li PC, Feng B. Advanced glycation end products increase lipids accumulation in macrophages through upregulation of receptor of advanced glycation end products: increasing uptake, esterification and decreasing efflux of cholesterol. Lipids Health Dis. 2016;15(1):161. 10.1186/s12944-016-0334-0 27644038 PMC5028926

[19] Tian K , Xu Y, Sahebkar A, Xu S. CD36 in atherosclerosis: pathophysiological mechanisms and therapeutic implications. Curr Atheroscler Rep. 2020;22(10):59. 10.1007/s11883-020-00870-8 32772254

[20] Zimmermann R , Panzenböck U, Wintersperger A, et al. Lipoprotein lipase mediates the uptake of glycated LDL in fibroblasts, endothelial cells, and macrophages. Diabetes. 2001;50(7):1643-1653. 10.2337/diabetes.50.7.1643 11423487

[21] Veiraiah A. Hyperglycemia, lipoprotein glycation, and vascular disease. Angiology. 2005;56(4):431-438. 10.1177/000331970505600411 16079928

[22] Jones NL , Reagan JW, Willingham MC. The pathogenesis of foam cell formation: modified LDL stimulates uptake of co-incubated LDL via macropinocytosis. Arterioscler Thromb Vasc Biol. 2000;20(3):773-781. 10.1161/01.atv.20.3.773 10712403

[23] Jha JC , Ho F, Dan C, Jandeleit-Dahm K. A causal link between oxidative stress and inflammation in cardiovascular and renal complications of diabetes. Clin Sci (Lond). 2018;132(16):1811-1836. 10.1042/CS20171459 30166499

[24] Loppnow H , Buerke M, Werdan K, Rose-John S. Contribution of vascular cell-derived cytokines to innate and inflammatory pathways in atherogenesis. J Cell Mol Med. 2011;15(3):484-500. 10.1111/j.1582-4934.2010.01245.x 21199323 PMC3922371

[25] Zhao Z , Zhang Z, Li J, et al. Sustained TNF-α stimulation leads to transcriptional memory that greatly enhances signal sensitivity and robustness. ELife. 2020;9(6):e61965. 10.7554/eLife.61965 33155547 PMC7704108

[26] Kamada R , Yang W, Zhang Y, et al. Interferon stimulation creates chromatin marks and establishes transcriptional memory. Proc Natl Acad Sci USA. 2018;115(39):E9162-E9171. 10.1073/pnas.1720930115 30201712 PMC6166839

[27] Macosko EZ , Basu A, Satija R, et al. Highly parallel genome-wide expression profiling of individual cells using nanoliter droplets. Cell. 2015;161(5):1202-1214. 10.1016/j.cell.2015.05.002 26000488 PMC4481139

[28] Trimm E , Red-Horse K. Vascular endothelial cell development and diversity. Nat Rev Cardiol. 2023;20(3):197-210. 10.1038/s41569-022-00770-1 36198871 PMC9533272

[29] Paik DT , Tian L, Lee J, et al. Large-scale single-cell RNA-Seq reveals molecular signatures of heterogeneous populations of human induced pluripotent stem cell-derived endothelial cells. Circ Res. 2018;123(4):443-450. 10.1161/CIRCRESAHA.118.312913 29986945 PMC6202208

[30] Zhang J , Chu LF, Hou Z, et al. Functional characterization of human pluripotent stem cell-derived arterial endothelial cells. Proc Natl Acad Sci USA. 2017;114(30):E6072-E6078. 10.1073/pnas.1702295114 28696312 PMC5544294

[31] Ang LT , Nguyen AT, Liu KJ, et al. Generating human artery and vein cells from pluripotent stem cells highlights the arterial tropism of Nipah and Hendra viruses. Cell. 2022;185(14):2523-2541.e30. 10.1016/j.cell.2022.05.024 35738284 PMC9617591

[32] Denny JB. Molecular mechanisms, biological actions, and neuropharmacology of the growth-associated protein GAP-43. Curr Neuropharmacol. 2006;4(4):293-304. 10.2174/157015906778520782 18654638 PMC2475799

[33] Zhou J , Wang L, Ling S, Zhang X. Expression changes of growth-associated protein-43 (GAP-43) and mitogen-activated protein kinase phosphatase-1 (MKP-1) and in hippocampus of streptozotocin-induced diabetic cognitive impairment rats. Exp Neurol. 2007;206(2):201-208. 10.1016/j.expneurol.2007.04.013 17601561

[34] Bursova S , Dubovy P, Vlckova-Moravcova E, et al. Expression of growth-associated protein 43 in the skin nerve fibers of patients with type 2 diabetes mellitus. J Neurol Sci. 2012;315(1-2):60-63. 10.1016/j.jns.2011.11.038 22209024

