# Supplementary material for: Unveiling impaired vascular function and cellular heterogeneity in diabetic donor-derived vascular organoids
Source: Stem Cells. 2024 Jul 25;42(9):791–808. doi: 10.1093/stmcls/sxae043 (PMC11384901; doi:10.1093/stmcls/sxae043)
Supplement: sxae043_suppl_Supplementary_Materials [file sxae043_suppl_supplementary_materials.zip › sxae043/Figures_Supps.pdf]

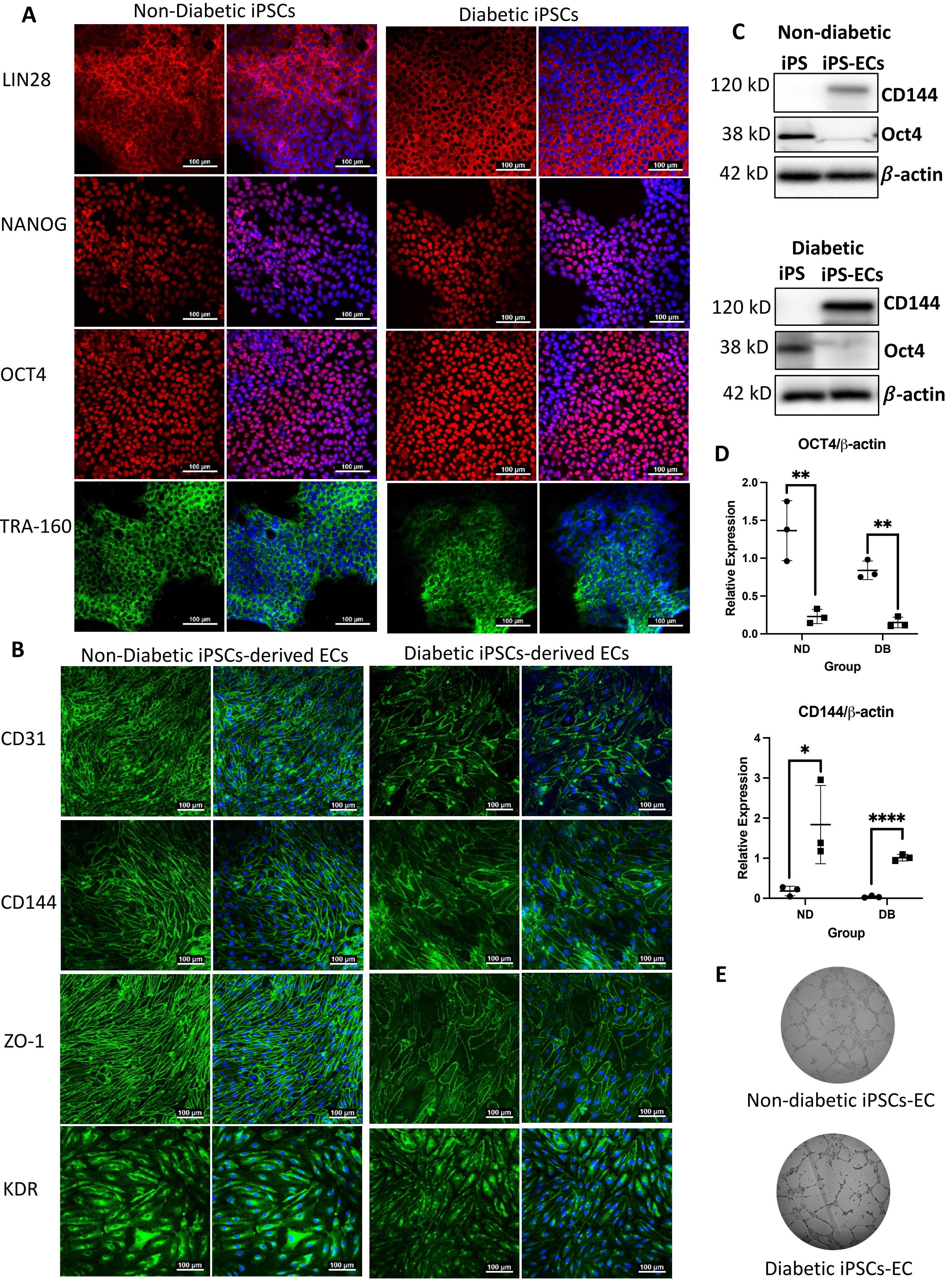
A
Non-Diabetic iPSCs
Diabetic iPSCs
LIN28
NANOG
OCT4
TRA-160
100 µm
B
Non-Diabetic iPSCs-derived ECs
Diabetic iPSCs-derived ECs
CD31
CD144
ZO-1
KDR
C
Non-diabetic
iPS
iPS-ECs
120 kD
38 kD
42 kD
CD144
Oct4
β-actin
Diabetic
iPS
iPS-ECs
120 kD
38 kD
42 kD
CD144
Oct4
β-actin
D
OCT4/β-actin
Relative Expression
2.0
1.5
1.0
0.5
0.0
**
**
ND
DB
Group
CD144/β-actin
Relative Expression
4
3
2
1
0
*
****
ND
DB
Group
E
Non-diabetic iPSCs-EC
Diabetic iPSCs-EC

Supplemental Figure 1. Characterization of human mononuclear blood cells-derived iPSCs and subsequent differentiation into iPS-EC.

A, Immunocytochemistry reveals the expression of pluripotency markers, including LIN28, NANOG, OCT4, and TRA-160, in reprogrammed cells from both non-diabetic (ND22) and diabetic (DB07) donors.

B, Subsequent differentiation of these cell lines into endothelial cells (iPSC-ECs) confirms the expression of endothelial markers such as CD31, CD144, ZO-1, and KDR. Scale bars represent 100 µm.

C, Expression of OCT4 in iPSCs and CD144 in iPS-ECs was further validated by Western blot, quantified from three independent lines of non-diabetic (ND05, ND19, ND22) and diabetic (DB07, DB13, and DB14) donors (**D**). In both cases, the analysis demonstrates the downregulation of pluripotency markers and the upregulation of endothelial markers in iPS-ECs compared to iPS cells. Data are presented as mean ± SEM; *$p < 0.05$, **$p < 0.01$, ***$p < 0.001$.

E, Tube formation assays confirm that both DB and ND iPSC-ECs were able to make endothelial tubes.

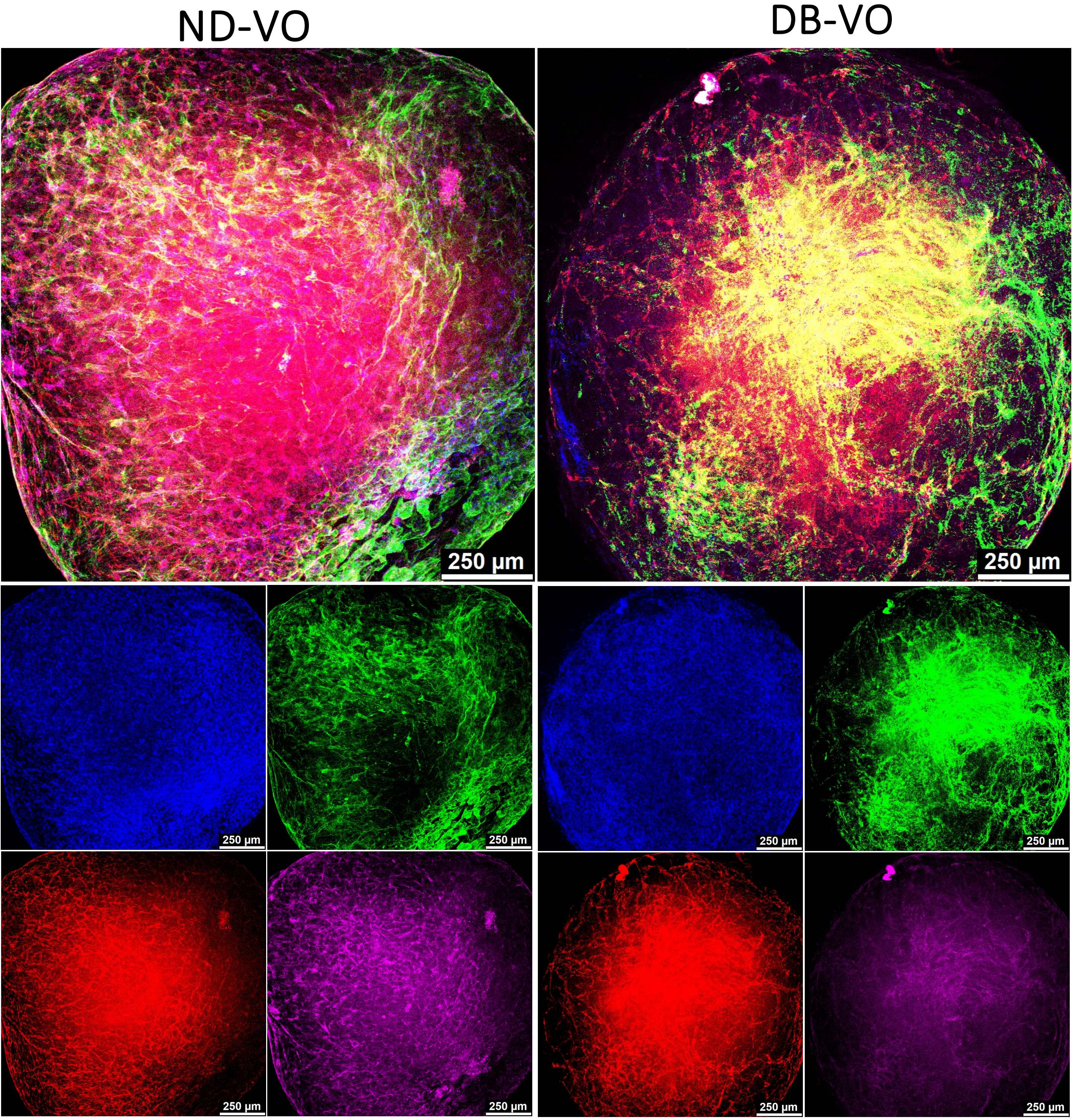

Supplemental Figure 2. Generation of Vascular organoids from iPSCs of both diabetic and non-diabetic individuals. Successful and reproducible generation of vascular organoids (VOs) from iPSCs of both diabetic (DB) and non-DB (ND) individuals. Confocal imaging showed the presence of both endothelial tubes (CD31^{+}, RED), mural cells (PDGFR-b^{+}, MAGENDA), and basement membrane (CollagenIV^{+}, GREEN) within the ND-VOs and DB-VOs. The upper panel is the merged image of its 4 separate channels in the lower panel. Blue is DAPI. Scale bar = 250µm. The size range of organoids was 0.5 to 1.5 mm.

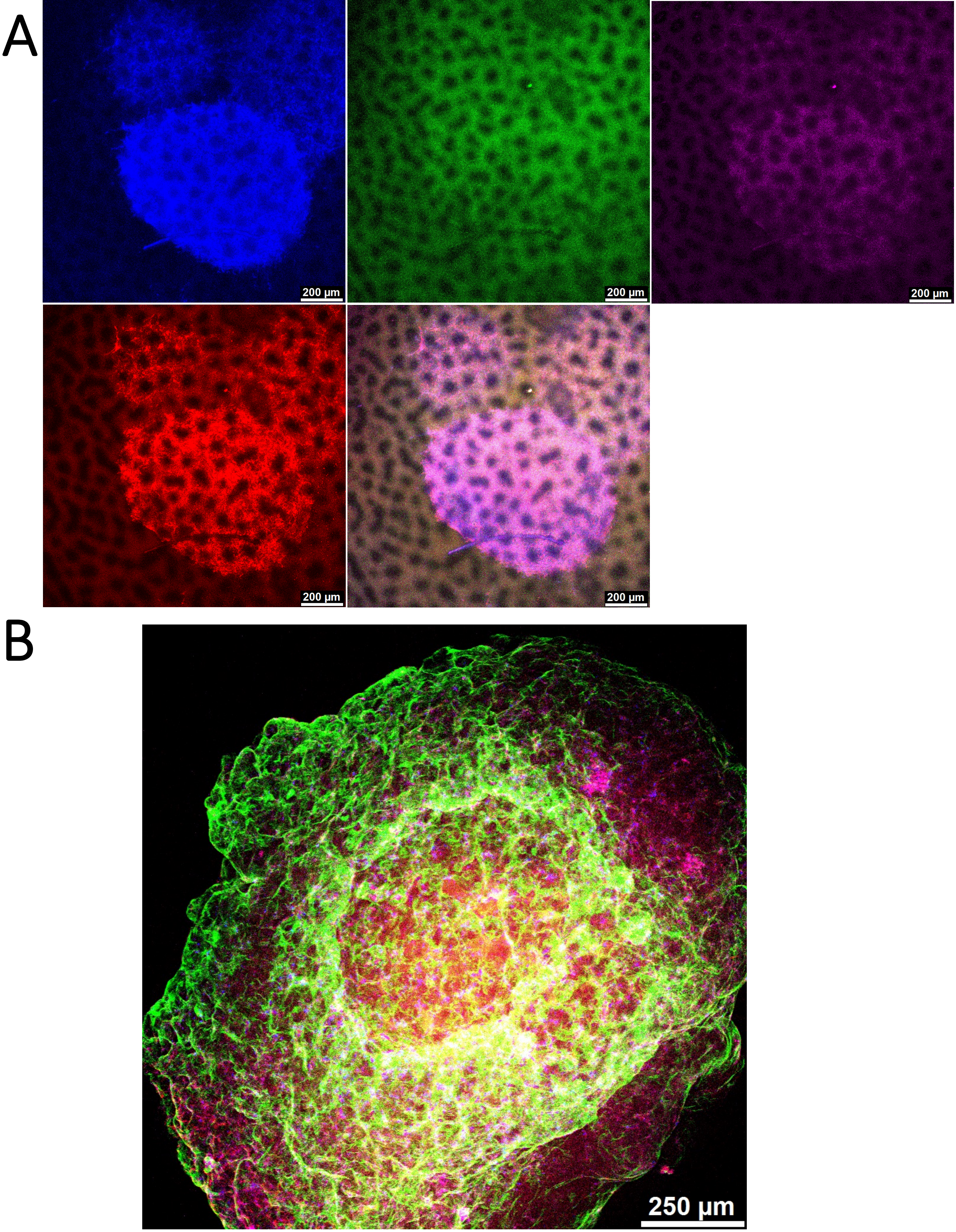

Supplemental Figure 3. Generation of vascular organoids mimics the embryonic pattern of vascular development. Primary plexus formation in vascular organoids (**A**) that subsequently remodel into a network of capillaries, arteries, and veins (**B**). Endothelial tubes (CD31^{+}, RED), mural cells (PDGFR-b^{+}, MAGENDA), and basement membrane (CollagenIV^{+}, GREEN).

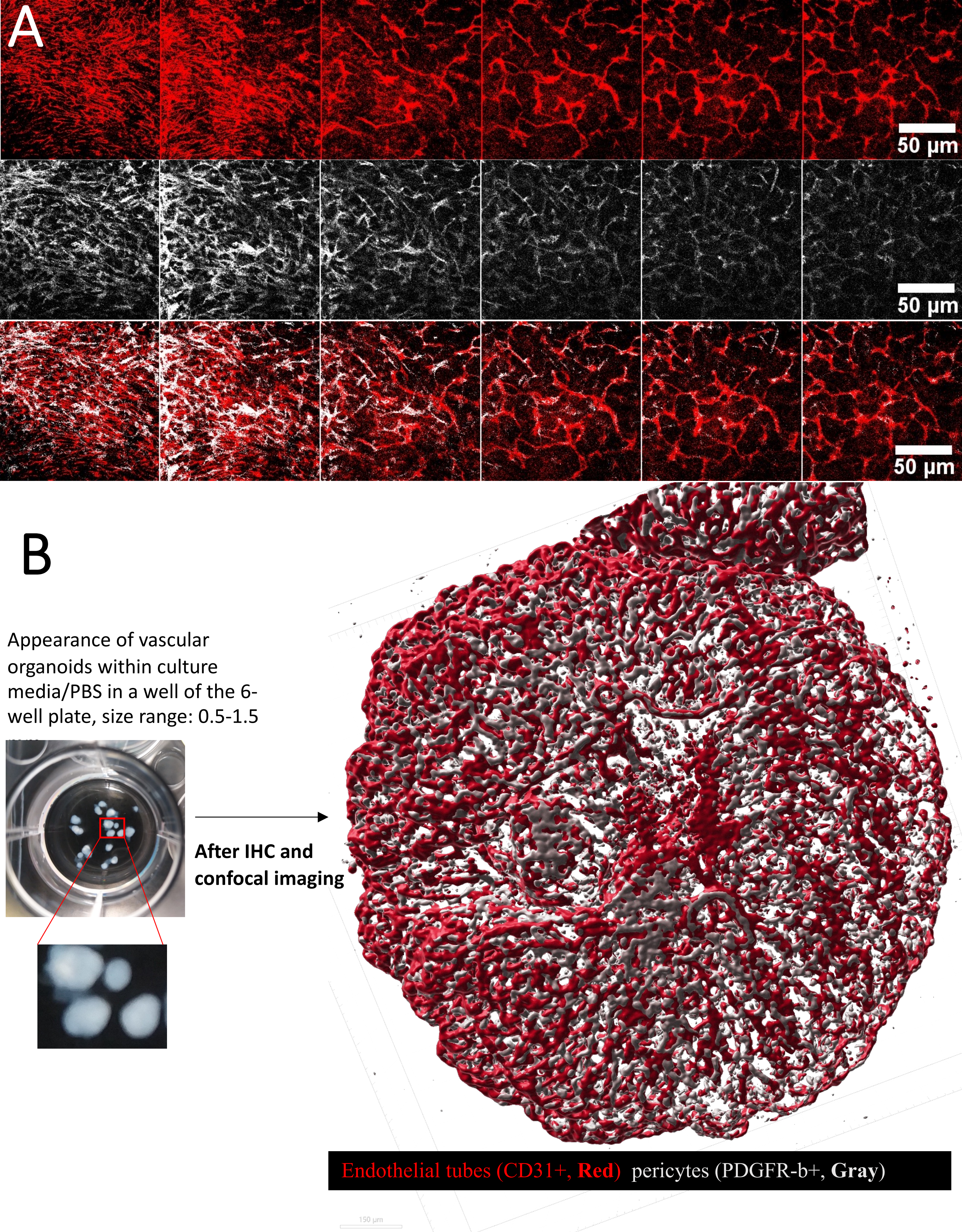

Supplemental Figure 4. Presence of both small and large vessels. A, Slices of confocal images showing endothelial cells and mural cells and their alignment together within vascular organoids within the same field of Z-stacks. Endothelial tubes (CD31+, Red), mural cells (PDGFR-b+, Gray). **B**, Appearance of 3-dimensional vascular organoids in a tissue culture plate and after confocal imaging; showing how nicely pericytes are covering endothelial tubes.

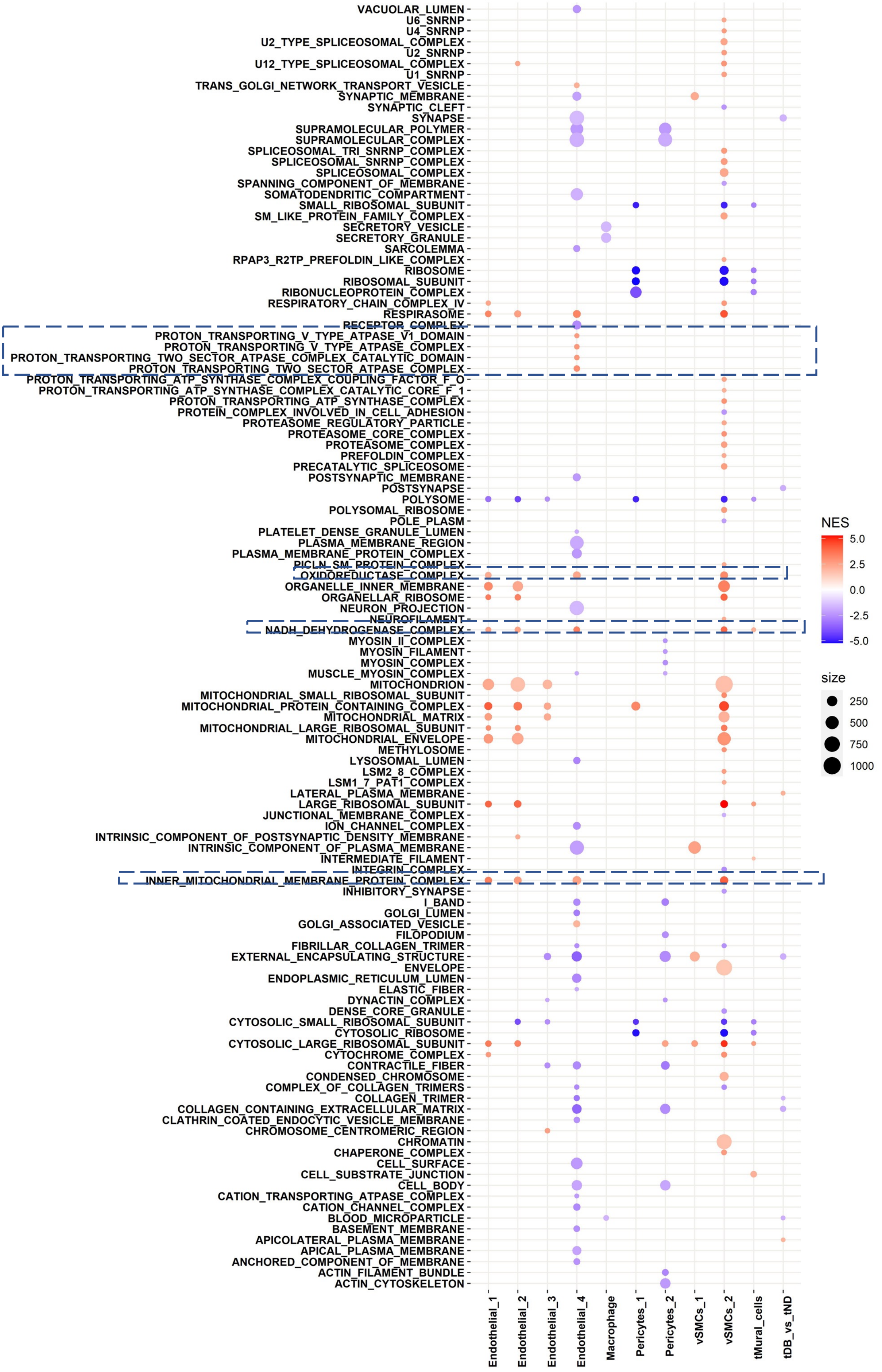

Supplemental Figure 5. Enrichment of diabetic vascular organoids for mitochondria. A bubble plot of only significant (at least in one condition) Gene Ontology-Cellular Components (**GO-CC**) has been shown out of a total of 999 CCs. Mitochondrial components are highly enriched in DB-VOs versus ND-VOs. FDR <0.01 was considered significant.

A

Array 1: CM mix of 3 DBs | CM mix of 3 NDs

Array 2: CM mix of 3 DBs | CM mix of 3 NDs

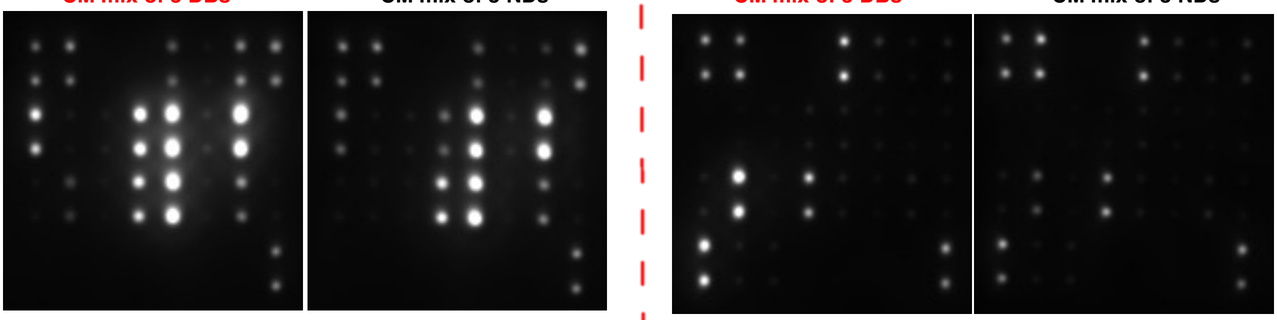

Fold change of signal intensity DBs vs. non-DBs, Array 1

Lin./Col.	A	B	C	D	E	F	G	H
L1	POS	POS	NEG	NEG	ANG	EGF	ENA-78	bFGF
L2					1.24		2.93	
L3	GRO	INF-g	IGF-1	IL-6	IL-8	Leptin	MCP1	PDGF-BB
L4	3.25			4.11	1.31	2.78	1.28	
L5	PLGF	RANTES	TGF-b1	TIMP-1	TIMP-2	TPO	VEGF-A	VEGF-D
L6		9.56		1.04	1.19		1.24	
L7	B	B	B	B	B	B	NEG	POS
L8								

Fold change of signal intensity DBs vs. non-DBs, Array 2

Lin./Col.	A	B	C	D	E	F	G	H
L1	POS	POS	NEG	NEG	ANGPT 1	ANGPT 2	Angiostatin	Endostatin
L2					1.65	2.39		
L3	GCSF	GM-CSF	CCL1	IL-10	IL-1a	IL-1b	IL-2	IL-4
L4					1.45	4.44		
L5	I-TAC	MCP-3	MCP4	MMP-1	MMP-9	CD31	Tie-2	TNF-a
L6		11.60		1.54				
L7	uPAR	VEGFR2	VEGFR3	B	B	B	B	POS
L8	3.39							

B

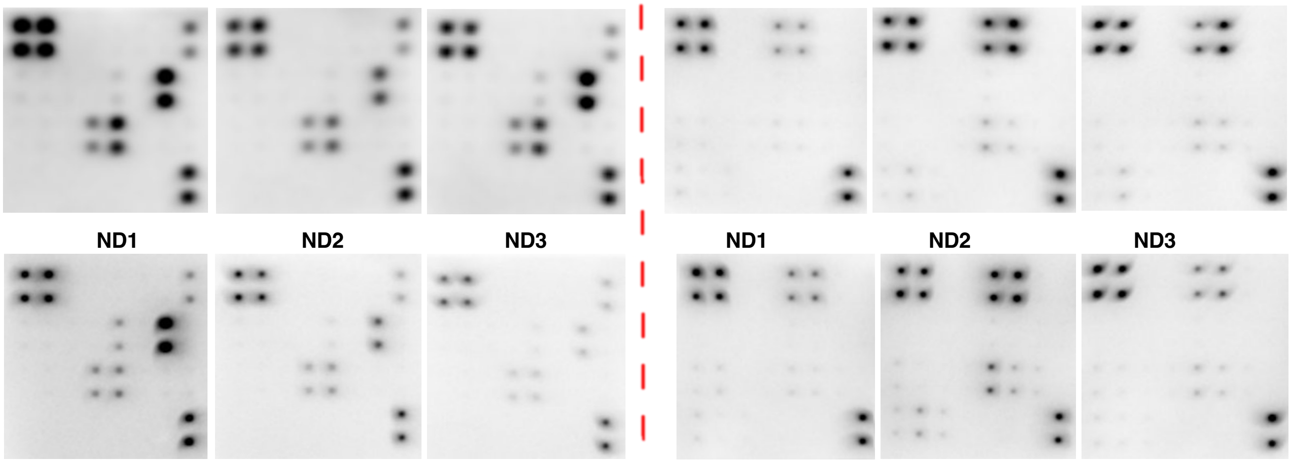

Average fold change of signal intensity DBs vs. non-DBs, Array 1

Lin./Col.	A	B	C	D	E	F	G	H
L1	POS	POS	NEG	NEG	ANG	EGF	ENA-78	bFGF
L2								-2.22
L3	GRO	INF-g	IGF-1	IL-6	IL-8	Leptin	MCP1	PDGF-BB
L4					1.7		-2.22	
L5	PLGF	RANTES	TGF-b1	TIMP-1	TIMP-2	TPO	VEGF-A	VEGF-D
L6				-1.86	-3.82			
L7	B	B	B	B	B	B	NEG	POS
L8								

Average fold change of signal intensity DBs vs. non-DBs, Array 2

Lin./Col.	A	B	C	D	E	F	G	H
L1	POS	POS	NEG	NEG	ANGPT 1	ANGPT 2	Angiostatin	Endostatin
L2					1.51	1.97		
L3	GCSF	GM-CSF	CCL1	IL-10	IL-1a	IL-1b	IL-2	IL-4
L4					-1.21			
L5	I-TAC	MCP-3	MCP4	MMP-1	MMP-9	CD31	Tie-2	TNF-a
L6	2.78	-2.04		-1.14	-1.62	-4.24	1.09	
L7	uPAR	VEGFR 2	VEGFR 3	B	B	B	B	POS
L8	2.04	-1.96	-2.49					

Supplemental Figure 6. Human protein array of diabetic and non-diabetic vascular organoids. A, Comparison of the conditioned media (minus FBS), 1.5 ml per well for 24 hours, the mixture of 3 DBs versus 3 NDs vascular organoids. DB-VOs were treated with g+TNFa+IL6 and control only with mannitol. **B**, Comparison of protein extracts from 3 independent diabetic vascular organoids versus 3 independent ND-VOs. Left panel: Array 1, and right panel: Array 2. Tables showing the layout of antibodies against 20 proteins on Array 1, and 23 proteins on Array 2, with values representing the fold change ratio of each protein in DBs versus NDs.

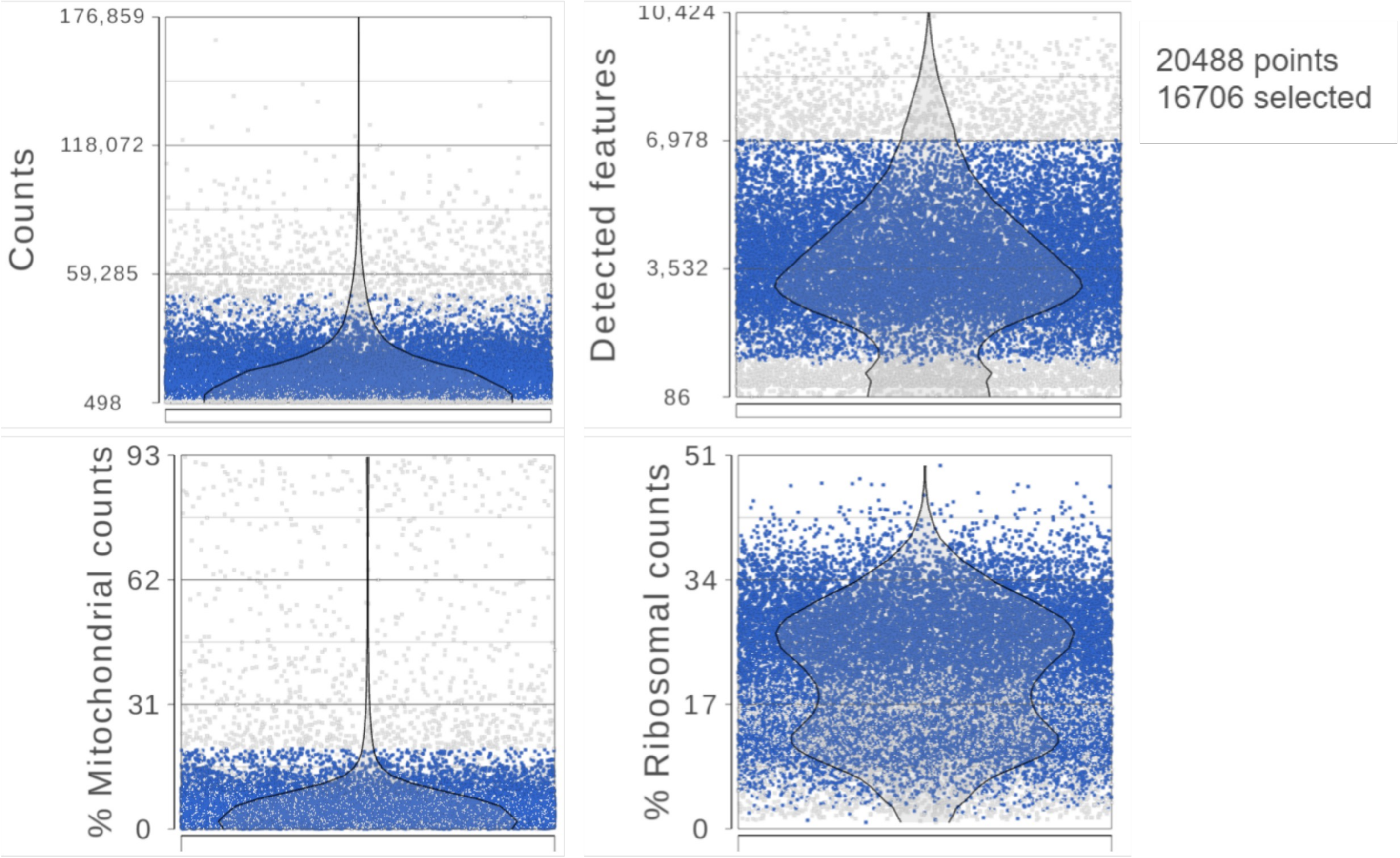

Sequencing output info (by cellranger-4.0.0)	NDs vascular organoids	DBs vascular organoids
Estimated Number of Cells	7,311	13,177
Median Genes per Cell	3,858	3,299
Total Genes Detected	24,822	25,994
Median UMI Counts per Cell	12,674	11,250
Mean Reads per Cell	59,335	43,968
Total Number of Reads	433,800,988	579,369,801

Supplemental Figure 7. Quality control trimming of single cell data. In the quality control step of single-cell data, cells with >7000 detected features (genes)/cell as the possible doublet/multiplet cells were excluded. Additionally, cells with <500 detected feature/cell and with low (<0.5%) or high (>50%) mitochondrial read counts as debris/low-quality cells (empty droplets containing apoptotic or dead cells' particles) filtered out. As a result, the remaining 16706 vascular organoids' cells (5799 ND and 10907 DB cells) out of a total of 20,488 cells), with a range of ≈500-60,000 unique molecular identifier counts/cell, were subjected to further downstream analysis.

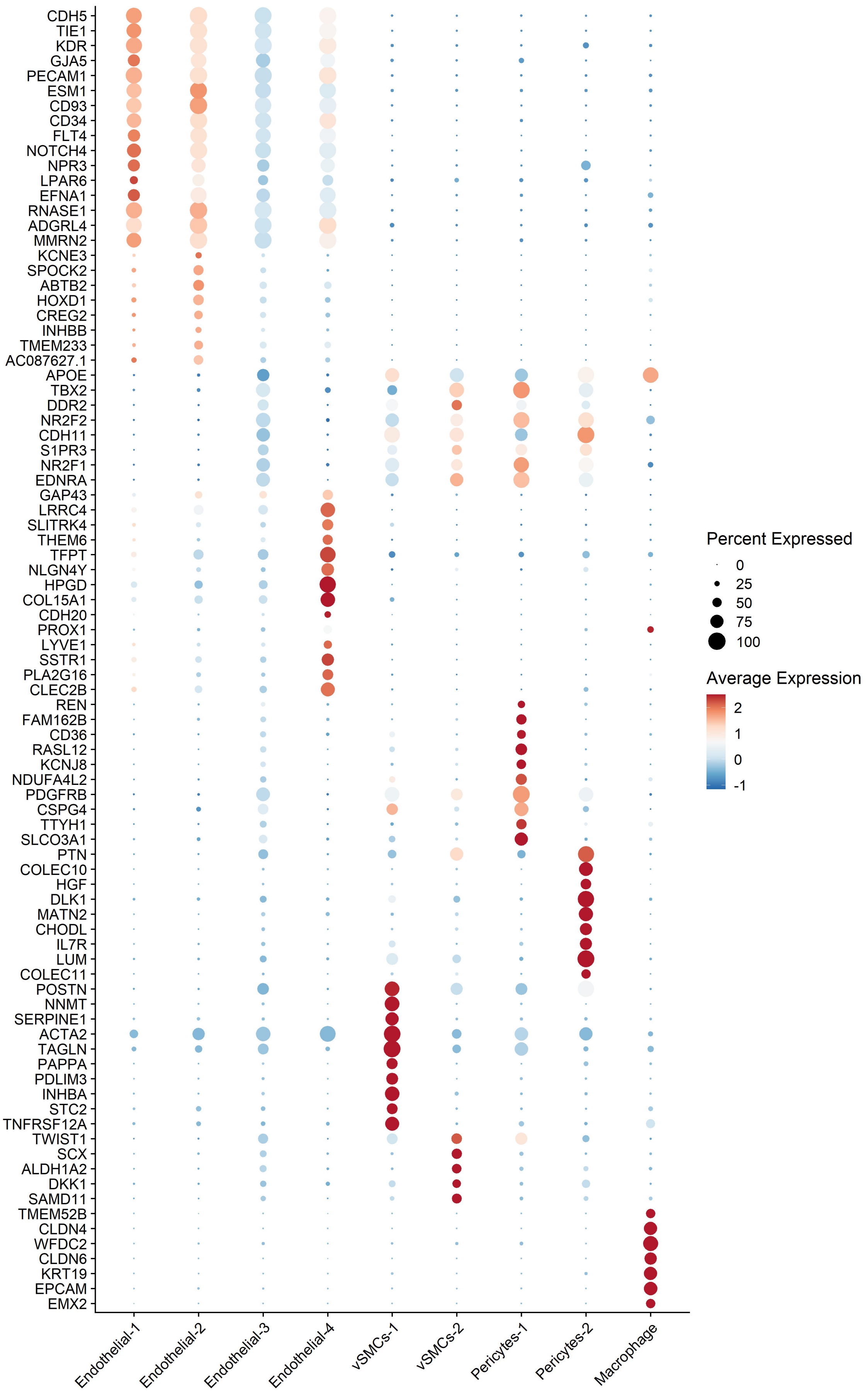

Supplemental Figure 8 Overview of some top biomarkers identified for each population within the vascular organoids. Biomarkers were calculated as differentially expressed genes (only positives) of each cell group against the rest of the cells, allowing for a more comprehensive understanding of the molecular profiles of these distinct cell populations. See the supplemental Excel Sheet for the full list of biomarkers.

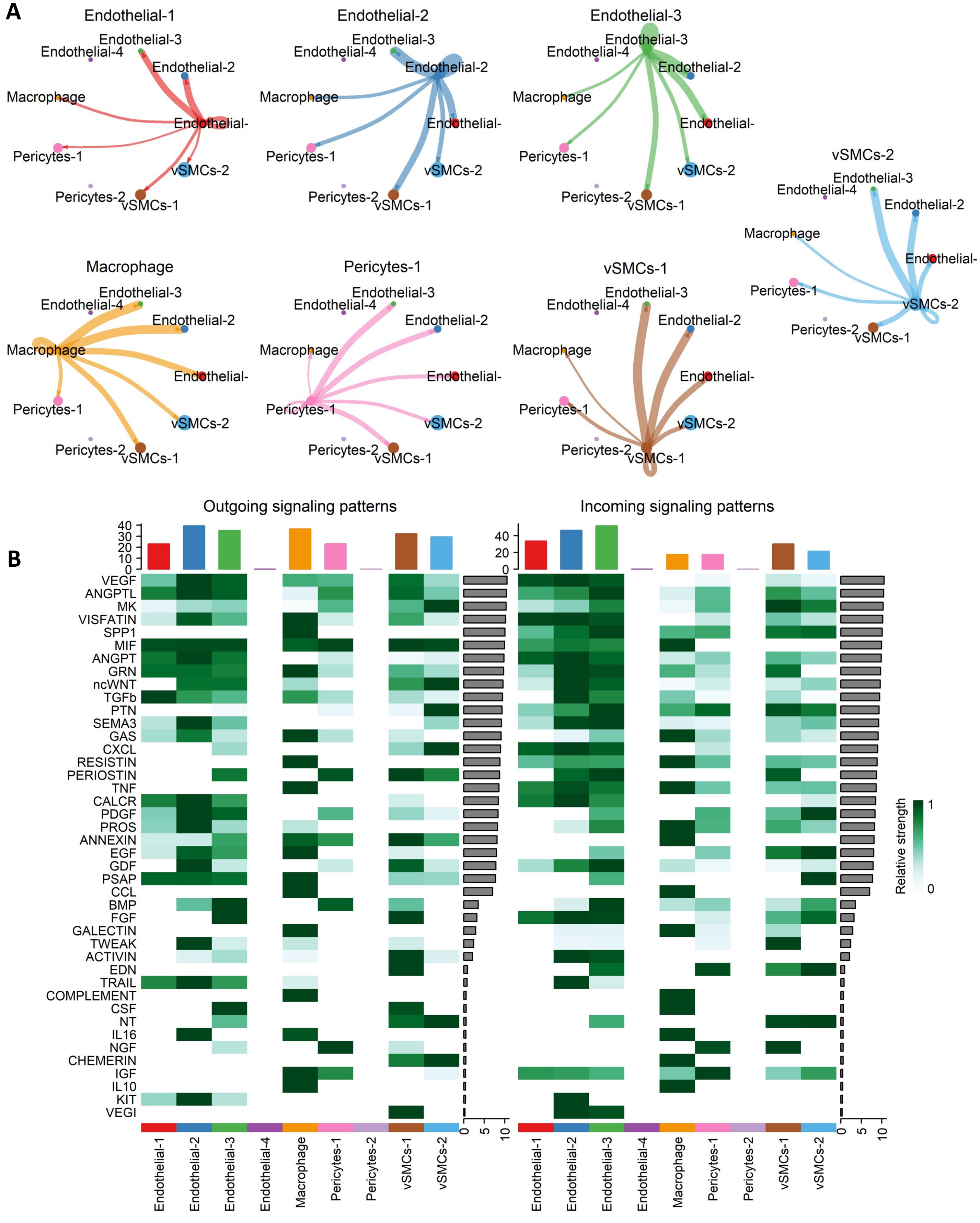

Supplemental Figure 9. Secreted signalling network within non-diabetic-donor iPSCs-derived vascular organoids containing endothelial and mural cells subpopulations. A, The graph visually represents the complex cell-cell communication network, allowing analysis of the signalling sent from each cell group. Nodes represent cell groups, and edges indicate signalling interactions, with thickness reflecting signalling strength. This graph aids the understanding of cellular communication and identifies potential signalling pathways in biological processes. **B**, The heatmap identifies important significant signalling molecules in the cell-cell communication network, highlighting potential targets for further investigation. By analyzing this heatmap, we can determine which signals contribute the most to the outgoing or incoming signalling of certain cell groups, providing a comprehensive view of the signalling roles played by various molecules. This understanding is critical for elucidating the complex mechanisms of cell-cell communication and their implications in diseases. The graphs were generated by the R package CellChat.

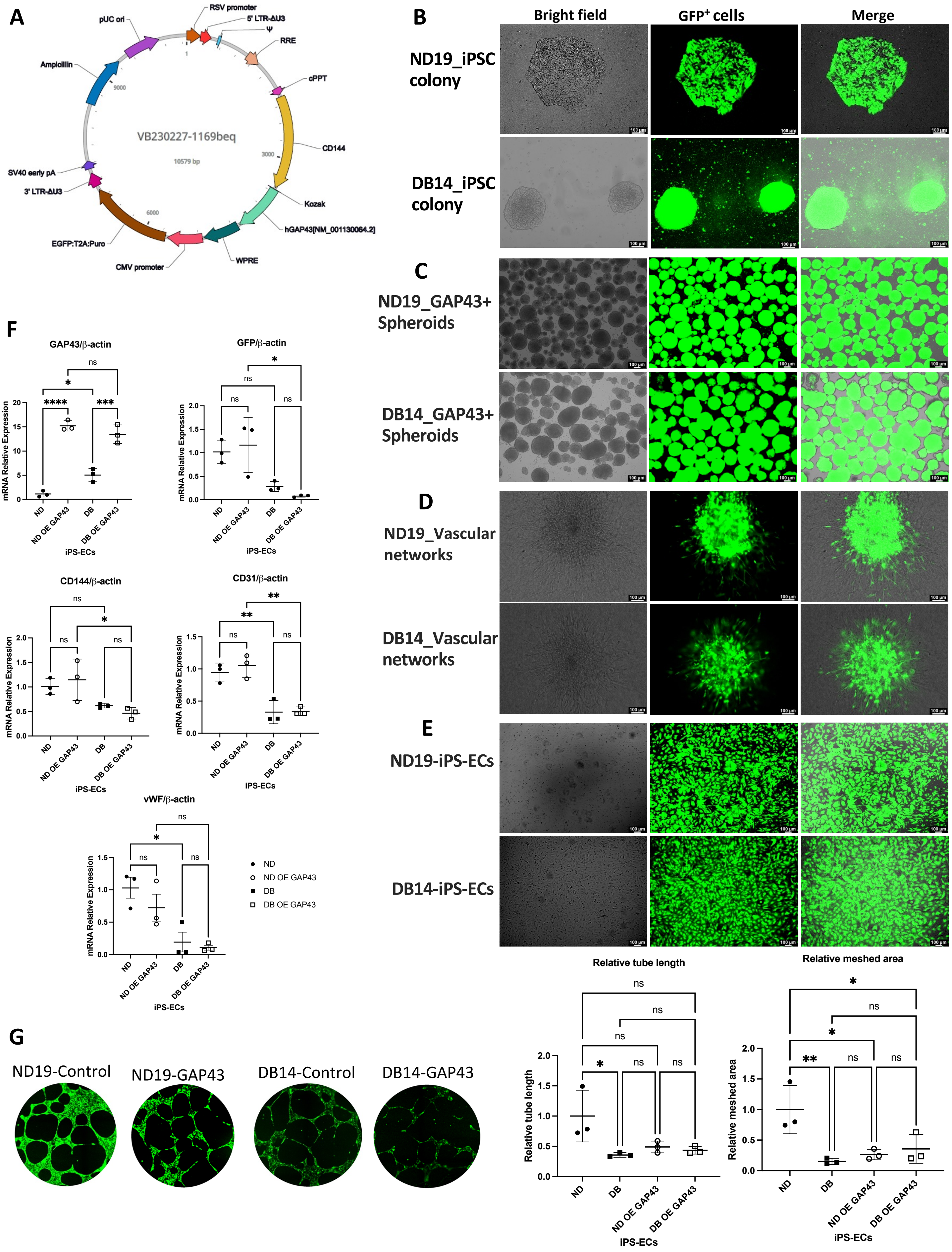
A
pUC ori
RSV promoter
5' LTR-ΔU3
Ψ
RRE
cPPT
Ampicillin
VB230227-1169beq
10579 bp
CD144
SV40 early pA
3' LTR-ΔU3
Kozak
hGAP43[NM_001130064.2]
EGFP:T2A:Puro
CMV promoter
WPRE
B
Bright field
GFP+ cells
Merge
ND19_iPSC colony
DB14_iPSC colony
C
ND19_GAP43+ Spheroids
DB14_GAP43+ Spheroids
D
ND19_Vascular networks
DB14_Vascular networks
E
ND19-iPS-ECs
DB14-iPS-ECs
F
GAP43/β-actin
GFP/β-actin
CD144/β-actin
CD31/β-actin
vWF/β-actin
mRNA Relative Expression
ND
ND OE GAP43
DB
DB OE GAP43
iPS-ECs
G
ND19-Control
ND19-GAP43
DB14-Control
DB14-GAP43
Relative tube length
Relative meshed area
iPS-ECs

Supplemental Figure 10. Effect of GAP43 overexpression in tube formation potential of ND-iPSECs and DB-iPSECs. A, GAP43 overexpression vector is designed under CD144 promotor to induce its expression in iPSECs but not in iPSCs. The same plasmid without GAP43 was used as vehicle control.
B, The colony of iPSCs (ND19 versus DB14) confirming uptake of the lentivector.
C, iPSCs-derived 3D spheroids (ND19 versus DB14) to induce differentiation into vascular networks.
D, endothelial tubes started to budding from the edge after induction of differentiation.
E, Purified iPS-ECs after selection by CD144 antibody by AutoMACS. Scale bars in all images of B-E = 100 μm.
F, RT-PCR confirmed expression of GAP43 and GFP relative to the iPSECs harbouring the control lentivector as well as expression of typical EC markers (CD31, CD144, and vWF).
G, Tube formation assay revealed that GAD43 overexpression (OE) has a negative impact in the function of iPSECs. Three technical replicates for each condition were quantified in bar plots in terms of relative meshed area and relative tube length.
