# Supplementary material for: Unveiling impaired vascular function and cellular heterogeneity in diabetic donor-derived vascular organoids
Source: Stem Cells. 2024 Jul 25;42(9):791–808. doi: 10.1093/stmcls/sxae043 (PMC11384901; doi:10.1093/stmcls/sxae043)
Supplement: sxae043_suppl_Supplementary_Materials [file sxae043_suppl_supplementary_materials.zip › sxae043/Supplemental Table 1_R1.docx]

**Supplemental Table 1:** Patient information from which iPS cells were derived and used in this study.

| Study ID | Age | Sex | Weight (kg) | Height (cm) | BMI | Blood Pressure (mmHg) | Diabetes | Diabetes duration |
| --- | --- | --- | --- | --- | --- | --- | --- | --- |
|  |  |  |  |  |  |  | type |  |
| **DB03** | 56 | M | 93 | 180 | 28.7 | 135/80 | Type 2 | 10-15 years |
| **DB04** | 85 | F | 61.3 | 153 | 26.1 | 145/75 | Type 2 | > 15 years |
| **DB07** | 72 | **F** | 63.5 | 147 | 29.4 | 160/70 | Type 2 | > 15 years |
| **DB09** | 63 | M | 141 | 164 | 52.4 | 125/67 | Type 2 | > 15 years |
| **DB11** | 60 | **F** | 99.1 | 152 | 42.8 | 156/61 | Type 2 | > 15 years |
| **DB12** | 89 | F | 72.2 | 151 | 31.6 | 134/81 | Type 2 | 5-10 years |
| **DB13** | 70 | M | 85.4 | 165 | 31.3 | 148/76 | Type 2 | > 15 years |
| **DB14** | 56 | **F** | 81.2 | 156 | 33.3 | 140/85 | Type 2 | 10-15 years |
| **ND05** | 59 | M | 88 | 185 | 25.7 | 115/80 | Not Diabetic | N/A |
| **ND19** | 58 | M | 92.9 | 181 | 28.3 | 136/83 | Not Diabetic | N/A |
| **ND20** | 71 | **F** | 67.7 | 159 | 26.7 | 139/64 | Not Diabetic | N/A |
| **ND21** | 61 | M | 71.8 | 161 | 27.5 | 119/80 | Not Diabetic | N/A |
| **ND22** | 58 | M | 65.4 | 163 | 24.6 | 131/74 | Not Diabetic | N/A |
